# The Catalase KatA Contributes to Microaerophilic H_2_O_2_ Priming to Acquire an Improved Oxidative Stress Resistance in *Staphylococcus aureus*

**DOI:** 10.3390/antiox11091793

**Published:** 2022-09-12

**Authors:** Nico Linzner, Vu Van Loi, Haike Antelmann

**Affiliations:** Institute of Biology-Microbiology, Freie Universität Berlin, 14195 Berlin, Germany

**Keywords:** *Staphylococcus aureus*, H_2_O_2_ resistance, priming, KatA, AhpC, Tpx, Bcp

## Abstract

*Staphylococcus aureus* has to cope with oxidative stress during infections. In this study, *S. aureus* was found to be resistant to 100 mM H_2_O_2_ during aerobic growth. While KatA was essential for this high aerobic H_2_O_2_ resistance, the peroxiredoxin AhpC contributed to detoxification of 0.4 mM H_2_O_2_ in the absence of KatA. In addition, the peroxiredoxins AhpC, Tpx and Bcp were found to be required for detoxification of cumene hydroperoxide (CHP). The high H_2_O_2_ tolerance of aerobic *S. aureus* cells was associated with priming by endogenous H_2_O_2_ levels, which was supported by an oxidative shift of the bacillithiol redox potential to −291 mV compared to −310 mV in microaerophilic cells. In contrast, *S. aureus* could be primed by sub-lethal doses of 100 µM H_2_O_2_ during microaerophilic growth to acquire an improved resistance towards the otherwise lethal triggering stimulus of 10 mM H_2_O_2_. This microaerophilic priming was dependent on increased KatA activity, whereas aerobic cells showed constitutive high KatA activity. Thus, KatA contributes to the high H_2_O_2_ resistance of aerobic cells and to microaerophilic H_2_O_2_ priming in order to survive the subsequent lethal triggering doses of H_2_O_2_, allowing the adaptation of *S. aureus* under infections to different oxygen environments.

## 1. Introduction

*Staphylococcus aureus* is a major human pathogen that can cause local skin and soft tissue infections, as well as life-threatening diseases, such as septicaemia, endocarditis, necrotizing pneumonia and osteomyelitis [1,2,3]. During infections, *S. aureus* has to cope with reactive oxygen species (ROS), such as superoxide anion (O_2_^•−^) and hydrogen peroxide (H_2_O_2_) [4], which are produced during the oxidative burst of activated macrophages and neutrophils to kill the invading pathogen [5,6,7,8]. The NADPH oxidase (NOX2) in the phagosomal membrane catalyses the one-electron transfer to molecular oxygen (O_2_) to generate O_2_^•−^, which is dismutated to H_2_O_2_ either spontaneously or by superoxide dismutases (SODs), including the extracellular SOD3 [6,8,9,10,11]. Both SOD3 and NOX2 are contained in secretory vesicles in macrophages and neutrophils and the fusion of these vesicles with phagosomes during phagocytosis might provide a mechanism for catalysed H_2_O_2_ production [11]. However, in neutrophils, the myeloperoxidase MPO is released from azurophilic granula into the phagosomal lumen, catalysing the dismutation of O_2_^•−^ to H_2_O_2_ upon infection [6]. MPO further converts H_2_O_2_ with chloride to the highly reactive hypochlorous acid (HOCl), which is the most potent oxidant and microbicidal agent released by activated neutrophils [6,9,10]. In addition, *S. aureus* encounters endogenous ROS during aerobic respiration due to the stepwise one-electron transfer reactions to O_2_, leading to production of O_2_^•−^ and H_2_O_2_ [12]. In the Fenton reaction, H_2_O_2_ reacts with free Fe^2+^ to generate the highly toxic hydroxyl radical (OH^•^), which can damage all cellular macromolecules, resulting in oxidation of proteins, lipids and carbohydrates [12,13,14]. However, H_2_O_2_ and HOCl can also function in redox signalling to activate or inactivate specific redox-sensitive regulators, which control defence mechanisms and confer resistance against the oxidants in bacterial pathogens [14,15].

*S. aureus* uses various enzymatic and non-enzymatic ROS and HOCl detoxification systems, such as a unique catalase (KatA), several peroxiredoxins (AhpC, Tpx, Bcp) and the low-molecular-weight (LMW) thiol bacillithiol (BSH) [14,16]. BSH associates with the bacilliredoxin/BSH/YpdA redox pathway to regenerate oxidized protein thiols and bacillithiol disulfide (BSSB) [16]. We previously constructed a Brx-roGFP2 fused biosensor to monitor the changes in the BSH redox potential (*E*_BSH_) under oxidative stress in *S. aureus* [17]. This study already revealed that *S. aureus* is highly resistant to 100 mM H_2_O_2_, since the Brx-roGFP2 biosensor responded only weakly to high H_2_O_2_ levels, leading to small *E*_BSH_ changes [17]. The catalase KatA was identified as the major H_2_O_2_ detoxification enzyme, which conferred the constitutive H_2_O_2_-resistant phenotype to aerobically grown *S. aureus* cells [18,19]. KatA is also important for nasal colonization and mediates protection under macrophage and neutrophil infections [18,20,21,22]. The peroxiredoxin AhpCF showed compensatory roles in resistance to H_2_O_2_ and organic hydroperoxides (OHPs) and contributed to nasal colonization [20]. OHPs (ROOH) are generated during oxidation of polyunsaturated fatty acids of eukaryotic membrane lipids and are reduced by peroxiredoxins to their corresponding organic alcohols [23].

In general, AhpC, Tpx and Bcp can be classified into typical (AhpC) or atypical (Tpx, Bcp) 2-Cys peroxiredoxins based on their thiol-oxidation mechanism between the peroxidatic (C_P_) and resolving Cys (C_R_), involving inter- or intramolecular disulfides, respectively [24]. The functions and substrates of AhpC, Tpx and Bcp have been previously studied in *Escherichia coli*. AhpC detoxification of H_2_O_2_ leads to formation of an oxidized AhpC dimer, which aggregates to an oligomer with chaperone functions [24,25]. Regeneration of AhpC requires the NADPH-dependent flavin disulfide reductase AhpF as a redox partner [24,25]. The thiol-peroxidase Bcp of *E. coli* is induced by H_2_O_2_, OHPs and during aerobic growth and confers resistance against H_2_O_2_ and OHP stress [26]. The thiol-peroxidase Tpx of *E. coli* has been shown to catalyse detoxification of H_2_O_2_ and OHPs in vitro and is recycled by the Trx/TrxR system [27,28]. In *S. aureus*, Tpx responds strongly to H_2_O_2_ and other thiol-reactive compounds and was oxidized in the redox proteome under HOCl stress [14,29]. However, the detailed functions of the peroxiredoxins AhpC, Tpx and Bcp in peroxide resistance, detoxification and survival have not been studied thus far in *S. aureus*.

In *S. aureus*, transcription of *katA*, *ahpCF* and *bcp* is strongly induced only by high levels of 10 mM H_2_O_2_ and controlled by the peroxide-responsive PerR repressor [18,20,30]. In *Bacillus subtilis*, KatA is also a member of the PerR regulon but already inducible by sub-lethal doses of 100 µM H_2_O_2_ [31,32]. Pretreatment of *B. subtilis* cells with sub-lethal H_2_O_2_ as “priming stimulus” confers improved resistance towards subsequent lethal H_2_O_2_ doses, termed as “triggering stimulus”, which are encountered as future stress [33,34]. These terms and abbreviations—priming (P), priming plus triggering (PT) and triggering (T)—were previously introduced within our project SFB973, which was directed to priming and memory of stress responses in different organisms, including bacteria, fungi and plants [35]. In *B. subtilis*, the H_2_O_2_ priming effect was shown to be mediated by KatA, which is induced by a mild stress to prepare the cells for better survival when faced with future lethal oxidative stress [33]. Similarly, H_2_O_2_ priming for improved resistance towards the triggering stimulus was dependent on the OxyR-dependent enzymes KatG and AhpCF in *E. coli* and *Salmonella* Typhimurium [32,36,37]. Although *S. aureus* exhibits constitutive H_2_O_2_ resistance during aerobic growth, it is unknown whether priming for improved H_2_O_2_ resistance is possible under aerobic or microaerophilic conditions.

In this study, we used growth and survival phenotype analyses, Brx-roGFP2 biosensor measurements and transcriptional studies to investigate the functions of KatA and the peroxiredoxins AhpC, Tpx and Bcp in peroxide resistance, detoxification and priming during aerobic and microaerophilic growth. Our results showed that *S. aureus* is H_2_O_2_ primable for improved resistance only under microaerophilic conditions, which are dependent on KatA. In contrast, aerobic growth already leads to increased levels of ROS, which causes KatA-dependent aerobic priming for constitutive H_2_O_2_ resistance. While KatA confers H_2_O_2_ resistance in *S. aureus*, the peroxiredoxins AhpC, Tpx and Bcp were shown to contribute to survival and resistance under CHP stress and regeneration of reduced *E*_BSH_ upon recovery from CHP stress.

## 2. Materials and Methods

### 2.1. Bacterial Strains, Growth and Survival Assays

Bacterial strains, plasmids and primers are described in Tables S1–S3. For genetic manipulation, *E. coli* was cultivated in Luria Broth (LB) medium. *S. aureus* COL strains were grown in RPMI medium to an optical density at 500 nm (OD_500_) of 0.5 and exposed to H_2_O_2_, cumene hydroperoxide (CHP) or hypochlorous acid (HOCl), followed by determination of colony-forming units (CFUs) in survival assays as previously described [38]. Each experiment was performed in at least three independent biological replicates and the results are presented as mean values with standard deviations (SD) from all biological replicates, as indicated in each figure legend. Statistical analysis was performed using Student’s unpaired two-tailed *t*-test with the software Graph Prism. The biochemical compounds were purchased from Sigma Aldrich. The HOCl concentration was determined as previously described [39].

### 2.2. Construction of the S. aureus COL ΔkatA, ΔahpC, ΔahpCΔkatA, Δtpx, Δbcp and ΔperR Mutants and Complemented Strains

The *S. aureus* COL Δ*katA* mutant and *katA* complemented strains were previously constructed [40]. The *S. aureus* Δ*ahpC*, Δ*tpx*, Δ*bcp* and Δ*perR* deletion mutants were constructed using the temperature-sensitive *E. coli-S.* *aureus* shuttle vector pMAD as previously described [41]. In brief, 500 bp of the up- and downstream flanking regions of the specific genes were fused by PCR, digested with *Bgl*II and *Sal*I and ligated into pMAD. The constructs were electroporated into the restriction-negative *S. aureus* RN4220, followed by phage transduction using phage 81 into *S. aureus* COL [42]. For construction of the Δ*ahpC*Δ*katA* double mutant, the plasmid pMAD-∆*katA* of *S. aureus* RN4220-pMAD-∆*katA* was transduced by the phage 81 into the *S. aureus* COL Δ*ahpC* mutant. Selection of the Δ*ahpC*, Δ*bcp*, Δ*tpx*, Δ*perR* and Δ*ahpC*Δ*katA* deletion mutants was performed as previously described [38].

Construction of the His-tagged *S. aureus ahpC*, *bcp* and *tpx* complemented strains was performed using the plasmid pRB473 as previously described [17]. The genes were cloned into pRB473 after digestion with *Bam*HI and *Kpn*I/*Sac*I, resulting in plasmids pRB473-*ahpC-His*, pRB473-*bcp-His* and pRB473-*tpx-His*, which were transduced in the Δ*ahpC*, Δ*bcp* and Δ*tpx* deletion mutants. In addition, the plasmid pRB473-*brx-roGFP2* [17] was introduced into the *S. aureus* COL Δ*katA*, Δ*ahpC*, Δ*ahpC*Δ*katA*, Δ*tpx* and Δ*bcp* deletion mutants to construct the Brx-roGFP2 biosensor expressing catalase- and peroxiredoxin-deficient mutant strains.

### 2.3. Priming and Triggering Experiments

For priming and triggering, the *S. aureus* strains were grown aerobically under vigorous agitation in shake flasks in a shaking water bath at 150 rpm or microaerophilically in 50 mL Falcon tubes including 40 mL cultures with closed lids without shaking, as in previous publications [43,44]. At an OD_500_ of 0.3, naïve *S. aureus* cells were primed by adding sub-lethal doses of 0.1 or 1 mM H_2_O_2_, respectively, to the bacterial culture for ~30 min. Subsequently, the lethal triggering doses of 10 or 40 mM H_2_O_2_, respectively, were added to the primed bacterial cultures, followed by counting of CFUs after 2 and 4 h of growth. For triggering only, naïve cells were treated with 10 or 40 mM H_2_O_2_ at an OD_500_ of 0.4, followed by counting of CFUs after 2 and 4 h of growth.

### 2.4. Brx-roGFP2 Biosensor Measurements

To monitor the *E*_BSH_ changes after H_2_O_2_ and CHP stress, we used the Brx-roGFP2 biosensor expressing WT, Δ*katA*, Δ*ahpC*, Δ*ahpC*Δ*katA*, Δ*tpx* and Δ*bcp* mutant strains and performed injection assays with the oxidants. For measurements of Brx-roGFP2 oxidation during microaerophilic and aerobic H_2_O_2_ priming and triggering experiments, the *S. aureus* COL strain expressing Brx-roGFP2 was cultivated in LB medium to an OD_540_ of 0.3 and challenged with the priming dose of 0.1 mM H_2_O_2_ for 30 min, followed by the triggering dose of 10 mM H_2_O_2_, as described above. Samples were harvested from *S. aureus* cells in the naïve (C), primed (P), primed and triggered (PT) and triggered-only (T) states, alkylated with 10 mM N-ethylmaleimide (NEM), washed and resuspended in PBS with 10 mM NEM. The Brx-roGFP2 oxidation degree (OxD) and *E*_BSH_ changes were determined in the *S. aureus* strains during oxidant injection or in samples harvested at C, P, PT and T as previously described [17,45]. For fully reduced and oxidized controls, biosensor strains were treated with 10 mM DTT and 5 mM diamide, respectively. The Brx-roGFP2 fluorescence emission was measured at 510 nm after excitation at 405 and 488 nm using the CLARIOstar microplate reader (BMG Labtech). The OxD of the Brx-roGFP2 biosensor was determined for each sample and normalized to fully reduced and oxidized controls as previously described [17,45].

### 2.5. Northern Blot Analyses

To analyze transcription of *katA*, *dps* and *ahpCF* in the *S. aureus* COL WT, Δ*katA*, Δ*ahpC* and Δ*perR* mutants using Northern blots, the *S. aureus* strains were grown in RPMI medium and harvested during the log phase at an OD_500_ of 0.4. To investigate *katA* and *ahpC* induction in the priming and triggering experiments, *S. aureus* WT cells were harvested in the naïve (C), primed (P), primed and triggered (PT) and triggered-only (T) states, as explained in the figure legends. RNA isolation was performed using the acid phenol extraction protocol as described previously [46]. Northern blot hybridizations were conducted using digoxigenin-labelled antisense RNA probes for *katA*, *ahpC* and *dps* that were synthesized in vitro using T7 RNA polymerase and the corresponding primers katA-NB-for/rev and ahpC-NB-for/rev, as previously described [46]. The *dps* antisense RNA probe was constructed previously [44].

### 2.6. Determination of the Catalase Activity Using Native PAGE and Diaminobenzidine Staining

To analyze catalase activities in *S. aureus* COL strains, protein extracts were prepared under native conditions and 50 µg of each sample was separated by non-denaturing 10% polyacrylamide gel electrophoresis. The gel was stained for catalase activity using 50 µg/mL horseradish peroxidase coupled with 5 mM H_2_O_2_ and 0.5 mg/mL diaminobenzidine, as described previously [47,48].

## 3. Results

### 3.1. S. aureus Exhibits KatA-Dependent H_2_O_2_ Resistance during Aerobic Growth

**Figure 1:**
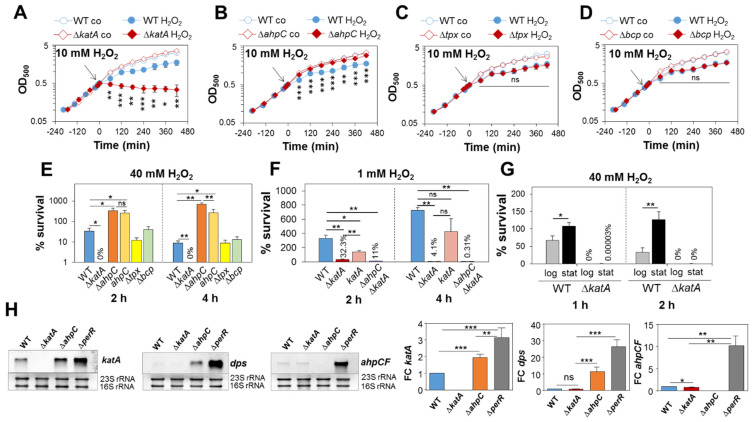
KatA confers strong H_2_O_2_ resistance during aerobic growth, while Tpx and Bcp are not required for H_2_O_2_ resistance. (**A**–**D**) Growth curves of *S. aureus* COL WT, Δ*katA* (**A**), Δ*ahpC* (**B**), Δ*tpx* (**C**) and Δ*bcp* mutants (**D**) in RPMI medium before (co) and after exposure to 10 mM H_2_O_2_ at an OD_500_ of 0.5. (**E**) Survival rates were determined as CFU counts for *S. aureus* COL WT, Δ*katA*, Δ*ahpC*, Δ*tpx* and Δ*bcp* mutants and the *ahpC* complemented strain at 2 and 4 h after treatment with 40 mM H_2_O_2_. (**F**) Survival rates were analysed for the WT, Δ*katA* and Δ*ahpC*Δ*katA* mutants and the *katA* complemented strain (*katA*) after 2 and 4 h of exposure to 1 mM H_2_O_2_ based on CFU counts. (**G**) *S. aureus* COL WT and Δ*katA* mutant cells were exposed to 40 mM H_2_O_2_ during the log and stationary phases at OD_500_ of 0.5 and 2–3, respectively. In (**E**–**G**), the survival rates were calculated relative to the untreated control, which was set to 100%. Mean values and standard deviation (SD) from three to four biological replicates are shown. (**H**) Northern blot analyses of the *katA*, *dps* and *ahpCF* specific transcripts in the *S. aureus* WT, Δ*katA*, Δ*ahpC* and Δ*perR* mutants. The methylene blue stains below the Northern blot images denote the bands of the 16S and 23S rRNAs used as RNA loading controls. Quantification of the intensities of the Northern blot bands was performed from two biological and three technical replicates using Image J and is shown in the diagrams as fold changes (FCs) of induction of the *katA*, *dps* and *ahpCF* transcripts in the mutants relative to the WT. Error bars represent the SD. The statistics were analysed using Student’s unpaired two-tailed *t*-test in Graph Prism. Symbols: ^ns^ *p* > 0.05, * *p* ≤ 0.05, ** *p* ≤ 0.01 and *** *p* ≤ 0.001.

To investigate the roles of the catalases and peroxiredoxins in the constitutive H_2_O_2_ resistance of *S. aureus* COL, phenotype analyses of the Δ*katA*, Δ*ahpC*, Δ*ahpC*Δ*katA*, Δ*tpx* and Δ*bcp* mutants and the complemented strains were performed during the aerobic growth under H_2_O_2_ stress (Figure 1; Figures S1 and S2). In agreement with previous findings [20], the Δ*katA* mutant was strongly impaired in growth after exposure to 10 mM H_2_O_2_ and did not survive doses of 40 mM H_2_O_2_ (Figure 1A,E). The *S. aureus* COL wild type (WT) was able to grow with low doses of 1 mM H_2_O_2_ and survived to 330 and 725% after 2 and 4 h, respectively (Figure 1F; Figure S1B). However, the Δ*katA* mutant was hypersensitive to peroxide stress, since the growth was inhibited by 0.4 and 1 mM H_2_O_2_ and only 32% and 4% of cells survived the 1 mM H_2_O_2_ treatment after 2 and 4 h, respectively (Figure 1F; Figure S1B). Complementation of the Δ*katA* mutant with pRB473-encoded *katA* could only partially restore the H_2_O_2_ resistance to WT level after treatment with 0.4–1 mM H_2_O_2_ (Figure 1F; Figure S1A–C). This incomplete recovery of the WT resistance was due to lower catalase activity in the *katA* complemented strain, as confirmed using the diaminobenzidine gel staining method (Figure S1D).

In addition, the H_2_O_2_ resistance of WT cells was 1.5–4-fold further enhanced during the stationary phase, whereas the Δ*katA* mutant did not survive 40 mM H_2_O_2_ during the log and stationary phases (Figure 1G). These data indicate that KatA also contributes to the stationary-phase H_2_O_2_ resistance in *S. aureus*. In contrast, the peroxiredoxin-deficient *S. aureus* Δ*tpx* and Δ*bcp* mutants were not impaired in growth or survival after exposure to 10 and 40 mM H_2_O_2_ (Figure 1C–E). However, the Δ*ahpC* mutant displayed an increased H_2_O_2_ resistance (Figure 1B,E), which was mediated by the PerR-dependent up-regulation of KatA in the Δ*ahpC* mutant, as shown previously [20]. To validate the derepression of PerR regulon genes in the Δ*ahpC* mutant, we analysed the transcription of *katA* and the miniferritin-encoding *dps* gene, since both were strongly up-regulated under different thiol-stress conditions in the transcriptome of *S. aureus* WT cells [43,49,50]. The Northern blot results revealed the twofold up-regulation of *katA*, while *dps* transcription was induced at an 11-fold higher rate in the Δ*ahpC* mutant, supporting the derepression of both PerR regulon genes in the Δ*ahpC* mutant under non-stress conditions (Figure 1H).

However, the H_2_O_2_-resistant phenotype of the Δ*ahpC* mutant could not be reversed to WT levels in growth and survival assays upon exposure to 10 and 40 mM H_2_O_2_ in the *ahpC* complemented strain (Figure 1E and Figure S2A). This lack of complementation might be caused by the lower plasmid-borne AhpC expression compared to the highly abundant AhpC in WT cells, as previously observed in the proteome [51]. The catalase activity was higher in the Δ*ahpC* mutant and *ahpC* complemented strain than in the WT, explaining the high H_2_O_2_ resistance upon complementation (Figure S2B). To confirm whether KatA and AhpC play additive roles in H_2_O_2_ resistance, the growth and survival of the Δ*ahpC*Δ*katA* double mutant was analysed. In agreement with previous data, the Δ*ahpC*Δ*katA* mutant showed a slower aerobic growth (Figure S3A) [20] and displayed 3-fold and 13-fold reduced survival rates after exposure to 1 mM H_2_O_2_ for 2 h and 4 h, respectively, as compared to the Δ*katA* mutant (Figure 1F).

Taken together, these results indicate that KatA plays the major role of conferring strong H_2_O_2_ resistance to aerobic *S. aureus* cells during the log and the stationary phases, whereas AhpC makes a minor contribution to the H_2_O_2_ resistance during the aerobic growth. Thus, KatA was identified as major determinant of the H_2_O_2_ resistance in growing and non-growing *S. aureus* cells.

### 3.2. The ΔkatA Mutant Shows a Strong Oxidative Shift in the E_BSH_ after H_2_O_2_ Stress and Is Impaired in Its Regeneration of the Reduced State, as Revealed by the Brx-roGFP2 Biosensor

**Figure 2:**
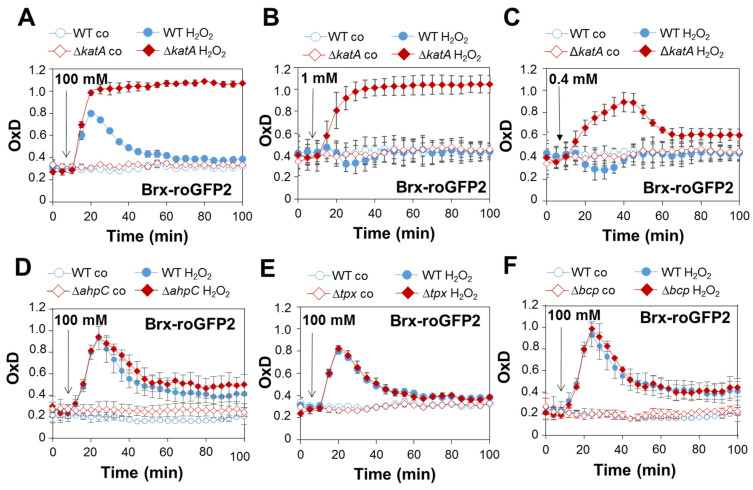
The Δ*katA* mutant can only detoxify 0.4 mM H_2_O_2_ stress, as revealed by the Brx-roGFP2 biosensor in *S. aureus*. (**A**–**F**) Brx-roGFP2 biosensor responses to 100, 1 and 0.4 mM H_2_O_2_ were monitored in the *S. aureus* COL WT, Δ*katA* (**A**–**C**), Δ*ahpC* (**D**), Δ*tpx* (**E**) and Δ*bcp* mutants (**F**) expressing Brx-roGFP2 from plasmid pRB473. H_2_O_2_ injection assays were performed in microplates using the CLARIOstar microplate reader, as described previously [45]. The oxidation degrees (OxD) of the Brx-roGFP2 responses were calculated based on the 405/488 nm excitation ratios and normalized to fully reduced (DTT-treated) and fully oxidized (diamide-treated) controls, as described previously [45]. Mean values and SD of the OxD values are presented from three independent biological replicates.

To monitor the changes in the *E*_BSH_ in the catalase- and peroxiredoxin-deficient mutants, we measured the Brx-roGFP2 biosensor responses after H_2_O_2_ stress in the WT and mutant strains. Due to the strong aerobic H_2_O_2_ resistance of *S. aureus* WT cells, the Brx-roGFP2 biosensor responded only weakly to 10 mM H_2_O_2_ in our previous studies [17]. Thus, we first used 100 H_2_O_2_ for WT cells, leading to fast biosensor oxidation and regeneration of the reduced state of *E*_BSH_ within two hours, as in our previous studies (Figure 2A) [17]. 

No increased biosensor oxidation was measured in WT cells after exposure to 1 mM H_2_O_2_ (Figure 2B). In contrast to WT cells, the biosensor was fully and constitutively oxidized by 1 and 100 mM H_2_O_2_ in the Δ*katA* mutant, indicated by an impaired regeneration of reduced *E*_BSH_ (Figure 2A,B). The *katA* mutant was only able to recover the reduced state of *E*_BSH_ after treatment with 0.4 mM H_2_O_2_ (Figure 2C), suggesting that this low H_2_O_2_ level might be detoxified by AhpC. In support of this hypothesis, the Brx-roGFP2 biosensor was quickly oxidized and strongly delayed in the recovery of reduced *E*_BSH_ after exposure to 0.4 mM H_2_O_2_ in the Δ*ahpC*Δ*katA* double mutant (Figure S3B). In contrast, the Δ*ahpC*, Δ*tpx* and Δ*bcp* mutants showed similar H_2_O_2_ responses and regeneration of reduced *E*_BSH_ compared to the WT (Figure 2D–F). The biosensor results confirmed the hypersensitivities of the Δ*katA* and Δ*ahpC*Δ*katA* mutants towards H_2_O_2_ stress, supporting that KatA was responsible for the rapid detoxification of 100 mM of H_2_O_2_ and regeneration of *E*_BSH_ in *S. aureus* WT cells, while AhpC could only detoxify low levels of 0.4 mM H_2_O_2_ in the absence of KatA. However, the peroxiredoxins Tpx and Bcp were not essential for H_2_O_2_ detoxification in *S. aureus* WT cells.

### 3.3. S. aureus Shows KatA-Dependent Microaerophilic H_2_O_2_ Priming to Acquire an Improved Resistance towards Lethal H_2_O_2_ Doses

**Figure 3:**
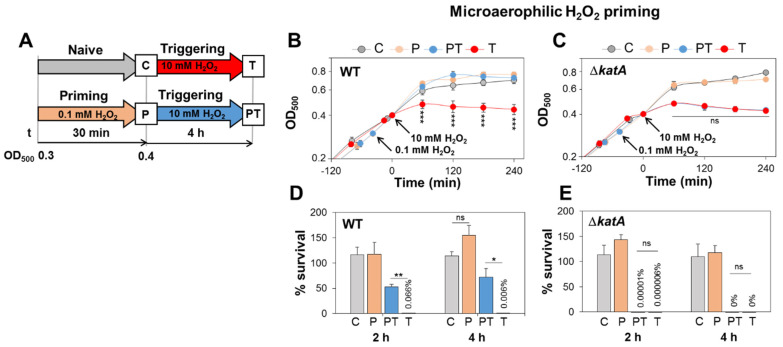
Microaerophilic H_2_O_2_ priming confers improved resistance against otherwise lethal H_2_O_2_, which depends on KatA. (**A**) Setup for microaerophilic priming and triggering experiments. The *S. aureus* WT and Δ*katA* mutant strains were grown microaerophilically and primed during the log phase with 0.1 mM H_2_O_2_ for ~30 min (P), followed by treatment with 10 mM H_2_O_2_ as a triggering stimulus (PT). The growth curves (**B**,**C**) and survival rates (**D**,**E**) were measured in naïve (**C**), primed (P), primed and triggered (PT) and triggered-only bacteria (T). The survival rates were calculated after 2 and 4 h of H_2_O_2_ stress relative to untreated control cells. The results are from three to four biological replicates. Error bars represent the SD. The statistics were calculated using Student’s unpaired two-tailed *t*-test in Graph Prism. Symbols: ^ns^ *p* > 0.05, * *p* < 0.05, ** *p* ≤ 0.01 and *** *p* ≤ 0.001.

The previous data revealed that KatA confers strong H_2_O_2_ resistance during aerobic growth. However, the role of KatA in the priming of *S. aureus* for improved H_2_O_2_ resistance during microaerophilic conditions was not investigated. Thus, the *S. aureus* WT and the Δ*katA* mutant were grown under microaerophilic conditions to the log phase and primed with 0.1 mM H_2_O_2_ for 30 min, followed by triggering with 10 mM H_2_O_2_ (Figure 3A). The growth and survival were analysed in naïve (C), primed (P), primed and triggered (PT) and triggered bacteria (T) (Figure 3A). 

The primed *S. aureus* WT and Δ*katA* mutant (P) were not impaired in growth and survival under 0.1 mM H_2_O_2_ (Figure 3B–E). However, the primed and triggered WT (PT) could acquire an improved resistance towards the triggering stimulus of 10 mM H_2_O_2_ compared to the triggering-only state (T) (Figure 3B,D). Specifically, PT bacteria showed survival rates of 52–72%, whereas T bacteria were almost killed and survived only to <0.07% after 10 mM H_2_O_2_ treatment. This indicates that *S. aureus* is primable for improved oxidative stress resistance during microaerophilic growth (Figure 3B,D). However, in contrast to the WT, the primed Δ*katA* mutant strain was unable to acquire the improved resistance towards otherwise lethal doses of 10 mM H_2_O_2_ under microaerophilic conditions (Figure 3C,E). Both PT and T bacteria of the Δ*katA* mutant were strongly impaired in growth and completely killed after treatment with 10 mM H_2_O_2_ as a triggering stimulus (Figure 3C,E).

However, due to its lower catalase activity from plasmid-based KatA expression (Figure S1D), the *katA* complemented strain did not recover the improved H_2_O_2_ resistance upon microaerophilic priming, resulting in growth inhibition and killing of PT and T bacteria (Figure S4). Overall, these results indicate that KatA is responsible for *S. aureus* priming for improved resistance against upcoming lethal H_2_O_2_ stress under microaerophilic conditions. Thus, KatA confers the constitutive resistance during aerobic growth and prepares *S. aureus* for future oxidative stress under microaerophilic conditions.

### 3.4. S. aureus Is Not Primable for Improved H_2_O_2_ Resistance during Aerobic Growth

**Figure 4:**
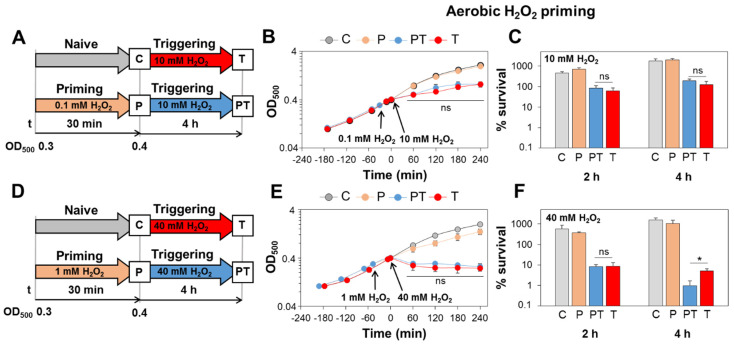
*S. aureus* is not primable for improved H_2_O_2_ resistance during aerobic growth. (**A**,**D**) Setup for aerobic priming and triggering experiments. The aerobically grown *S. aureus* WT was primed during the log phase with either 0.1 mM H_2_O_2_ (**A**) or 1 mM H_2_O_2_ (**D**) for ~30 min (P) and subsequently treated with either 10 mM H_2_O_2_ (**A**) or 40 mM H_2_O_2_ (**D**), respectively, as triggering stimuli (PT). The growth curves (**B**,**E**) and survival rates (**C**,**F**) were measured for naïve (**C**), primed (P), primed and triggered (PT) and triggered-only bacteria (T). Survival rates of the H_2_O_2_-treated cells were calculated relative to the untreated control. The results are from three to four biological replicates. Error bars represent the SD. The statistics were calculated using Student’s unpaired two-tailed *t*-test in Graph Prism. Symbols: ^ns^ *p* > 0.05 and * *p* < 0.05.

Next, priming and triggering experiments were performed in aerobically grown *S. aureus* cells to analyse whether the constitutive H_2_O_2_ resistance could be further enhanced in primed cells (Figure 4). First, we used the same H_2_O_2_ doses for the priming (0.1 mM) and triggering (10 mM) experiments as applied in the microaerophilic experiments (Figure 4A). As expected, there were no differences in growth and survival between PT and T bacteria after exposure to 10 mM H_2_O_2_ during the aerobic growth (Figure 4B,C). Both PT and T bacteria were similarly resistant and fully survived the 10 mM H_2_O_2_ triggering dose. Thus, the constitutive resistance of aerobic *S. aureus* cells towards 10 mM H_2_O_2_ could not be further enhanced by pre-exposure to the priming stimulus of 0.1 mM H_2_O_2_ (Figure 4B,C). As shown before, this constitutive H_2_O_2_ resistance of aerobically grown *S. aureus* cells was dependent on KatA (Figure 1A,E,F).

We further increased the H_2_O_2_ doses for priming (1 mM H_2_O_2_) and triggering (40 mM) of *S. aureus* during the aerobic growth (Figure 4D). However, higher priming doses also could not improve the growth and survival of PT bacteria in response to the subsequent 40 mM H_2_O_2_ stress compared to the T bacteria treated with 40 mM H_2_O_2_ only (Figure 4E,F). Both PT and T bacteria were strongly impaired in growth under 40 mM H_2_O_2_ stress and showed survival rates of <10% after 2 h.

Small survival differences of 4% were observed after 4 h in T versus PT bacteria but not at the 2 h time point (Figure 4F). In conclusion, these different priming setups support that *S. aureus* is not primable for enhanced H_2_O_2_ resistance under aerobic conditions. We hypothesize that ROS production during aerobic respiration acts as a priming stimulus to induce KatA, which confers the high H_2_O_2_ resistance.

### 3.5. Microaerophilic H_2_O_2_ Priming Causes Increased Transcription of KatA and Elevated KatA Activity, which Confers Improved Resistance towards Lethal H_2_O_2_ Doses in S. aureus

**Figure 5:**
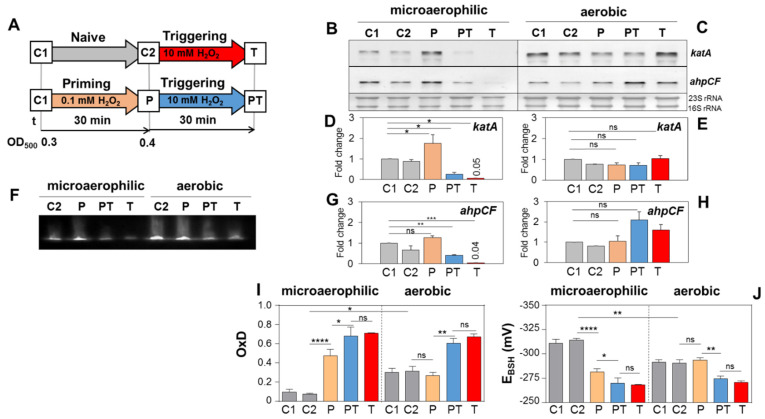
Microaerophilic H_2_O_2_ priming leads to increased *katA* transcription and KatA activity, as well as fast Brx-roGFP2 biosensor oxidation. (**A**) The setup for microaerophilic and aerobic priming and triggering experiments included priming with 0.1 mM H_2_O_2_ and triggering with 10 mM H_2_O_2_. (**B**,**C**) To analyze transcription of *katA* and *ahpCF* using Northern blots, RNA was isolated from *S. aureus* WT cells in the naïve (C1, C2), primed (P), primed and triggered (PT) and triggered (T) states. The band intensities of the *katA* (**D**,**E**) and *ahpCF* specific transcripts (**G**,**H**) were quantified from two biological replicates using ImageJ. The transcriptional induction of *katA* and *ahpCF* was calculated as fold change relative to the control C1, which was set to 1. Error bars represent the SD. The statistics were calculated using ordinary one-way ANOVA and Dunnet’s multiple comparisons test in Graph Prism. Symbols: ^ns^ *p* > 0.05; * *p* ≤ 0.05; ** *p* ≤ 0.01 and *** *p*≤ 0.001. (**F**) The catalase activity was analysed in cell extracts of the *S. aureus* WT during microaerophilic and aerobic H_2_O_2_ priming for C2, P, PT and T states using native PAGE and diaminobenzidine staining. The catalase activity assays were performed in two biological and two technical replicates. (**I**,**J**) The response of the Brx-roGFP2 biosensor was measured in *S. aureus* COL grown in LB medium under microaerophilic and aerobic conditions in naïve (C1, C2), primed (P), primed and triggered (PT) and triggered (T) cells. The C1 and C2 samples were harvested at OD_500_ of 0.3 and 0.4, respectively, and the P, PT and T bacteria were harvested after 10 min of H_2_O_2_ exposure (maximum biosensor oxidation). Samples were blocked with 10 mM NEM and the fluorescence excitation maxima were measured at 405 and 488 nm using the microplate reader. OxD values and the *E*_BSH_ of Brx-roGFP2 were calculated using the 405/408 nm excitation ratio, as described in the Section 2. Mean values of three biological replicates are shown, error bars represent the SD and *p*-values were calculated using Student’s unpaired two-tailed *t*-test in Graph Prism software. Symbols: ^ns^ *p* > 0.05; * *p* ≤0.05; ** *p* ≤ 0.01; *** *p* ≤ 0.001 and **** *p* ≤ 0.0001.

Northern blot analyses were used to study whether microaerophilic H_2_O_2_ priming induces transcription of the PerR-dependent *katA* gene and the *ahpCF* operon in *S. aureus* (Figure 5). The results revealed that transcription of *katA* was significantly up-regulated by 1.8-fold upon microaerophilic priming with 0.1 mM H_2_O_2_ (Figure 5B,D), whereas the basal level of *katA* transcription was already higher under aerobic conditions and could not be further induced during aerobic H_2_O_2_ priming (Figure 5C,E). Transcription of the *ahpCF* operon was not significantly induced during microaerophilic priming (Figure 5B,G). However, transcription of *katA* and *ahpCF* was strongly reduced after triggering by 10 mM H_2_O_2_ under microaerophilic conditions, since the triggering dose was lethal (Figure 5B,D,G). Transcription of *katA* decreased even in the PT bacteria compared to P cells, which highlights the high efficiency of the KatA protein for fast removal of H_2_O_2_ in PT bacteria (Figure 5B,D). Under aerobic conditions, PT and T bacteria did not show significantly enhanced transcription of *katA* and *ahpCF*, supporting that the constitutive H_2_O_2_ resistance could not be further enhanced by pre-exposure to the priming dose (Figure 5C,E,H).

The *katA* transcript levels could be confirmed by catalase activities during the microaerophilic and aerobic priming experiments (Figure 5F). Specifically, the basal activity of KatA was very low during the microaerophilic growth, whereas aerobic cells showed a constitutive high catalase activity. The KatA activity could be only enhanced upon microaerophilic priming but not during aerobic priming due to the high constitutive resistance (Figure 5F). Further consistent with the Northern blots, the KatA activity decreased strongly in PT and T bacteria during microaerophilic priming. Together, these results reveal that microaerophilic priming induces *katA* transcription and KatA activity, which confers improved resistance against otherwise lethal H_2_O_2_ stress.

### 3.6. Microaerophilic H_2_O_2_ Priming Leads to a Strong Oxidative Shift in the E_BSH_

We were interested in the *E*_BSH_ differences between aerobic and microaerophilic growth conditions, supporting the enhanced ROS levels in aerobic cells as endogenous priming stimuli. Moreover, we aimed to analyse the Brx-roGFP2 biosensor response upon microaerophilic priming and triggering to investigate if the KatA induction upon microaerophilic priming is accompanied by a change in the Brx-roGFP2 biosensor oxidation. The comparison of the basal biosensor oxidation revealed a more reducing basal OxD of 0.1 and an *E*_BSH_ of −310 mV during microaerophilic growth (C1) compared to the OxD of 0.3 and the *E*_BSH_ of −291 mV during aerobic growth (C1) (Figure 5I,J). Thus, the higher basal oxidation in aerobic cells accounted for the increased ROS level due to aerobic respiration. Upon microaerophilic priming, the Brx-roGFP2 biosensor showed a fivefold increased OxD and an oxidized *E*_BSH_ of −281 mV, which was further oxidized in PT and T bacteria. In contrast, the biosensor did not respond to aerobic priming and showed increased oxidation only in aerobic PT and T bacteria (Figure 5I,J). These results support that microaerophilic priming leads to an oxidative shift in the *E*_BSH_ from −310 mV to −281 mV, resulting in increased catalase expression that primes the cells for improved H_2_O_2_ resistance. Due to the higher basal oxidation in aerobic cells, priming did not change the high *E*_BSH_ of −291 mV, which is consistent with the constitutive KatA expression (Figure 5I,J). These results on the *E*_BSH_ differences of ~20 mV between microaerophilic and aerobic cells strongly support that respiratory H_2_O_2_ primes aerobic cells for constitutive H_2_O_2_ resistance.

### 3.7. The Peroxiredoxins AhpC, Tpx and Bcp Mediate CHP Resistance in S. aureus

**Figure 6:**
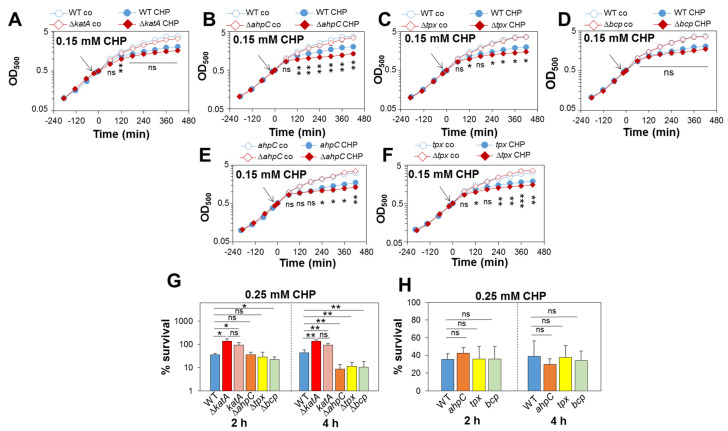
The peroxiredoxins AhpC, Tpx and Bcp contribute to CHP resistance in *S. aureus*. (**A**–**F**) Growth phenotypes were analysed for the *S. aureus* COL WT, Δ*katA* (**A**), Δ*ahpC* (**B**), Δ*tpx* (**C**) and Δ*bcp* mutants (**D**) and the *ahpC* (**E**) and *tpx* (**F**) complemented strains in RPMI medium before (co) and after exposure to 0.15 mM CHP stress at an OD_500_ of 0.5. (**G**,**H**) The survival rates of the *S. aureus* COL WT, Δ*katA*, Δ*ahpC*, Δ*tpx* and Δ*bcp* mutants (**G**) and the *katA* (**G**), *ahpC*, *tpx* and *bcp* (**H**) complemented strains were determined at 2 and 4 h after exposure to 0.25 mM CHP relative to the untreated control, which was set to 100%. Mean values and SD of four to six biological replicates are presented. The statistics were calculated using Student’s unpaired two-tailed *t*-test in Graph Prism. Symbols: ^ns^ *p* > 0.05, * *p* ≤ 0.05, ** *p* ≤ 0.01 and *** *p* ≤ 0.001.

While the role of KatA in aerobic H_2_O_2_ resistance and microaerophilic H_2_O_2_ priming was clearly revealed, the peroxiredoxins AhpC, Tpx and Bcp could also function in the resistance to organic hydroperoxides in *S. aureus*. Using growth and survival assays, the phenotypes of the catalase and peroxiredoxin-deficient mutants were analysed after CHP treatment (Figure 6). While the growth of the Δ*katA* and Δ*bcp* mutants was not affected by 0.15 mM CHP stress, the CHP-treated Δ*ahpC* and Δ*tpx* mutants showed slightly reduced growth rates (Figure 6A–D), which could be restored to WT levels upon complementation (Figure 6E,F). In addition, the Δ*ahpC*, Δ*tpx* and Δ*bcp* mutants showed significantly decreased survival rates after 4 h of CHP stress, supporting that the peroxiredoxins confer protection against CHP stress in *S. aureus* (Figure 6G). These CHP-sensitive survival phenotypes of the peroxiredoxin-deficient mutants could be restored to WT levels in the *ahpC*, *tpx* and *bcp* complemented strains (Figure 6H). However, the slightly increased CHP resistance of the Δ*katA* mutant could not be reverted to WT levels in the *katA* complemented strain (Figure 6G), probably due to the partial complementation by plasmid-based KatA expression (Figure S1D).

### 3.8. Peroxiredoxin-Deficient ΔahpC, Δtpx and Δbcp Mutants Are Delayed in CHP Detoxification as Revealed by Brx-roGFP2 Measurements

**Figure 7:**
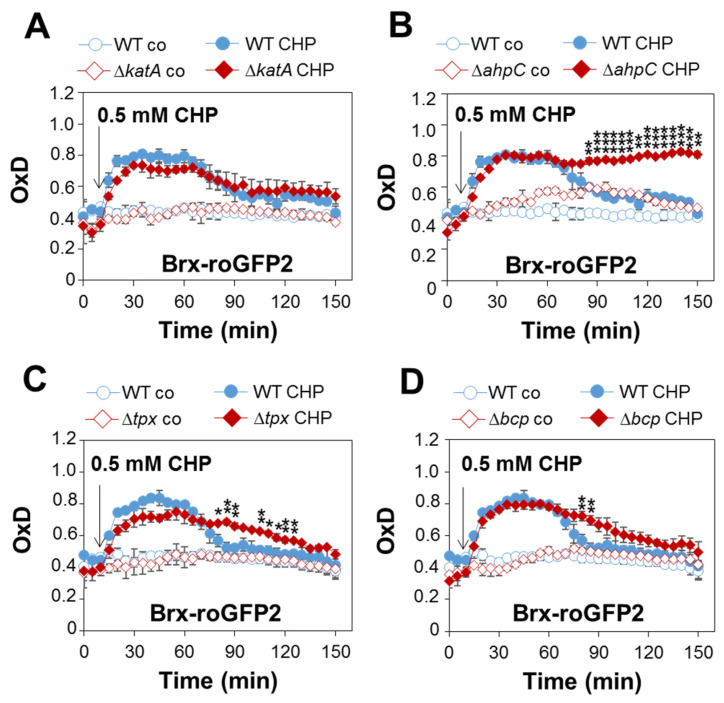
AhpC, Tpx and Bcp function in CHP detoxification and regeneration of reduced *E*_BSH_ during the recovery phase, as revealed by the Brx-roGFP2 biosensor. (**A**–**D**) Brx-roGFP2 responses to 0.5 mM CHP in *S. aureus* COL WT, Δ*katA* (**A**), Δ*ahpC* (**B**), Δ*tpx* (**C**) and Δ*bcp* mutants expressing Brx-roGFP2 from plasmid pRB473. H_2_O_2_ injection assays were performed in microplates and the biosensor responses measured using the CLARIOstar microplate reader, as described previously [45]. (**D**) The oxidation degree (OxD) of the Brx-roGFP2 response was calculated based on the 405/488 nm excitation ratio and normalized to fully reduced (DTT-treated) and oxidized (diamide-treated) controls. Mean values and SD of three biological replicates are shown. The statistics were calculated using Student’s unpaired two-tailed *t*-test in Graph Prism. Symbols: ^ns^ *p* > 0.05, * *p* ≤ 0.05, ** *p* ≤ 0.01 and *** *p* ≤ 0.001.

To investigate the impact of the peroxiredoxins in the maintenance of the reduced state of *E*_BSH_ in *S. aureus*, Brx-roGFP2 biosensor measurements were performed after 0.5 mM CHP stress (Figure 7). The Brx-roGFP2 biosensor was similarly quickly oxidized by 0.5 mM CHP in the WT, Δ*katA*, Δ*ahpC*, Δ*tpx* and Δ*bcp* mutants. However, while the WT and Δ*katA* mutant could regenerate the reduced state of *E*_BSH_ within 2 h, the Δ*ahpC* mutant was unable to regenerate the basal level of *E*_BSH_ during the recovery phase from CHP stress (Figure 7A,B). In addition, both the Δ*bcp* and Δ*tpx* mutants showed significant delays in recovery of the reduced state of *E*_BSH_ upon CHP stress as compared to the WT (Figure 7C,D). These biosensor results support that the peroxiredoxins AhpC, Tpx and Bcp are important for CHP detoxification and contribute to regeneration of *E*_BSH_ during the recovery phase from CHP stress in *S. aureus*. In contrast, KatA does not contribute to CHP detoxification and resistance in *S. aureus.*

### 3.9. KatA and Peroxiredoxins Do Not Contribute to Protection against HOCl Stress

Previous transcriptome analyses revealed an increased transcription of the PerR regulon under HOCl stress in *S. aureus* [38]. Thus, growth curves and survival assays were used to analyse the phenotypes of the Δ*katA*, Δ*ahpC*, Δ*tpx* and Δ*bcp* mutants under HOCl stress. However, none of these mutants showed significant defects in growth or survival after HOCl stress compared to the WT, indicating that the catalase and peroxiredoxins do not contribute to HOCl detoxification and resistance in *S. aureus* (Figure S5A–E).

## 4. Discussion

Here, we investigated the roles of catalase and peroxiredoxins in the peroxide stress resistance and priming of *S. aureus* during aerobic and microaerophilic growth. Our results revealed that *S. aureus* is not primable towards improved H_2_O_2_ resistance during aerobic growth due to its constitutive H_2_O_2_ resistance, which was shown to be dependent on the catalase KatA. Moreover, we found that microaerophilic *S. aureus* cells can be primed to acquire an enhanced resistance towards lethal H_2_O_2_ doses, which was also mediated by KatA (Figure 3B,D). In addition, the peroxiredoxins AhpC, Tpx and Bcp were shown to contribute to the CHP detoxification and resistance in *S. aureus* to ensure the maintenance of the redox balance upon recovery from stress.

The roles of KatA and AhpC in the peroxide resistance of *S. aureus* have been previously demonstrated [20]. In this work, we additionally used Brx-roGFP2 biosensor measurements, showing the impact of KatA and AhpC on the level of H_2_O_2_ detoxification and regeneration of reduced *E*_BSH_ under oxidative stress. Without KatA, *S. aureus* cells are highly sensitive to oxidants and only able to remove low doses of 0.4 mM H_2_O_2_, which were shown to be detoxified by AhpC. In addition, the Δ*ahpC*Δ*katA* double mutant showed an increased sensitivity towards H_2_O_2_ stress in survival assays as compared to the Δ*katA* mutant. These data confirm that KatA and AhpCF have compensatory roles in H_2_O_2_ resistance to ensure the survival of *S. aureus* under oxidative stress [20]. Expression of *katA*, *bcp*, *dps* and the *ahpCF* operon is controlled by the peroxide-sensing PerR repressor, which is inactivated by H_2_O_2_ due to Fe^2+^-catalysed histidine oxidation in *S. aureus* [18,52,53]. Due to PerR derepression in the Δ*ahpC* mutant, *katA* and *dps e*xpression was elevated, as confirmed here using Northern blots and shown previously [20]. Thus, the higher KatA expression level in the Δ*ahpC* mutant mediates the enhanced H_2_O_2_ resistance, confirming previous results in *S. aureus* and *B. subtilis* [20,54,55].

In addition, we showed that aerobically grown *S. aureus* acquire an improved resistance to H_2_O_2_ during the stationary phase, which also depends on KatA. The oxidative stress resistance was also enhanced during the stationary phase in other bacteria, such as *B. subtilis* and *E. coli* [56,57]. In addition, KatA activity was elevated during the stationary phase in *S. aureus* and *B. subtilis* [20,48]. Altogether, our results on aerobic *S. aureus* cells reveal that KatA is the major player that confers the strong constitutive H_2_O_2_ resistance during the log and stationary phases, while the peroxiredoxin AhpC plays an additional role of scavenging 0.4 mM H_2_O_2_ in the absence of KatA. Apart from its major role as an H_2_O_2_ scavenger, the catalase also provides heme and iron as cofactors for cellular metabolism, which could contribute to *S. aureus* survival under oxidative stress.

**Figure 8:**
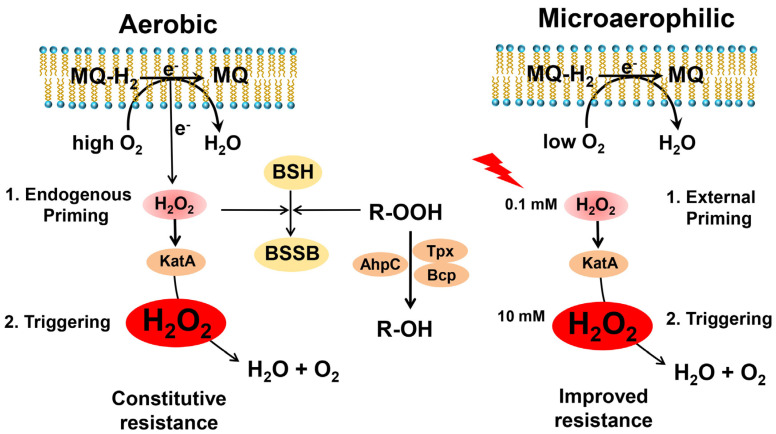
Summary schematics of microaerophilic H_2_O_2_ priming (**right**) and constitutive aerobic H_2_O_2_ resistance (**left**) in *S. aureus*. We found that microaerophilic priming with 100 µM H_2_O_2_ induces improved resistance towards otherwise lethal doses of 10 mM H_2_O_2_ in *S. aureus* (**right**), while aerobic cells are already primed by endogenous ROS originating from respiration, resulting in the constitutive high H_2_O_2_ resistance (**left**). The higher ROS levels during the aerobic growth were revealed by the ~20 mV increased basal *E*_BSH_ compared to microaerophilic cells. The catalase KatA was shown to be induced upon microaerophilic H_2_O_2_ priming (**right**) and by endogenous H_2_O_2_ during aerobic respiration (**left**), mediating improved and constitutive H_2_O_2_ resistance, respectively, in *S. aureus*. In addition, the peroxiredoxins AhpC, Tpx and Bcp were shown to confer resistance towards organic hydroperoxides (R-OOH). Furthermore, KatA and the peroxiredoxins contribute to the regeneration of reduced *E*_BSH_ upon recovery from oxidative stress. MQ-H_2_ and MQ indicate reduced and oxidized menaquinone. BSH and BSSB are reduced bacillithiol and oxidized bacillithiol disulfide, respectively.

We further showed that *S. aureus* is primable with sub-lethal doses of 0.1 mM H_2_O_2_ to acquire an improved resistance towards otherwise lethal doses of 10 mM H_2_O_2_ during the microaerophilic growth. This microaerophilic H_2_O_2_ priming was found to be dependent on KatA, which was transcriptionally induced and showed a higher catalase activity upon challenge with the priming dose. These results are consistent with previous data showing increased KatA activity by exposure to 100 µM H_2_O_2_ during oxygen limitation [52]. We showed that microaerophilic priming by KatA prepares the cells to better survive the lethal triggering stress, resulting in improved growth and survival of *S. aureus.* In contrast, due to their constitutive H_2_O_2_ resistance, aerobic *S. aureus* cells were not primable to acquire higher resistance towards lethal H_2_O_2_ doses. Aerobic priming was not possible with 0.1 mM or 1 mM H_2_O_2_ since *katA* transcription and catalase activity were already elevated in naïve cells and could not be further increased in primed cells. The higher basal level of *E*_BSH_ of −291 mV in aerobic cells compared to the more reducing basal *E*_BSH_ of −310 mV in microaerophilic cells strongly indicates an increased ROS level in aerobic cells due to aerobic respiration. These biosensor data support that *S. aureus* is primed by endogenous ROS generated by aerobic respiration to achieve their constitutive H_2_O_2_ resistance phenotype (Figure 8). Thus, the PerR regulon is already up-regulated during aerobic growth due to ROS generated during aerobic respiration [52], resulting in the H_2_O_2_-resistant phenotype. 

This up-regulation of the PerR regulon in *S. aureus* during the aerobic growth was caused by the hypersensitive PerR repressor, which is poised by very low endogenous levels of H_2_O_2_ generated during aerobic respiration [52]. The endogenous H_2_O_2_ concentration in aerobic *E. coli* cells was determined as ~50 nM [58]. While PerR of *S. aureus* is hypersensitive to endogenous H_2_O_2_ levels during aerobic growth, the PerR protein of *B. subtilis* is less sensitive and cannot sense such low H_2_O_2_ levels originating from aerobic respiration [52]. Thus, *B. subtilis* can be primed during aerobic growth with 0.1 mM H_2_O_2_, leading to PerR inactivation and derepression of the PerR-controlled KatA, which confers an adaptive resistance against the otherwise lethal triggering dose of 10 mM H_2_O_2_ [33,34,52,55,59].

Similarly, aerobic priming is possible in *E. coli* and *S*. Typhimurium after exposure to 100 µM H_2_O_2_, leading to activation of the OxyR regulon, including the major catalase, which confers an adaptive and improved resistance towards lethal concentrations of 10 mM H_2_O_2_ [36,57,60]. In conclusion, priming of bacteria towards improved H_2_O_2_ resistance depends on the sensitivity of the redox-sensing peroxide regulators. While many bacteria harbour less sensitive H_2_O_2_ sensors and are primable by sub-lethal H_2_O_2_ doses during aerobic growth, *S. aureus* PerR is hypersensitive to endogenous H_2_O_2_ levels during aerobic growth [52], resulting in constitutive aerobic H_2_O_2_ resistance and microaerophilic priming to acquire improved H_2_O_2_ resistance only in oxygen-limited conditions (Figure 8).

While KatA is responsible for detoxification of up to 100 mM H_2_O_2_ and confers the high resistance to 100 mM H_2_O_2_ in aerobic *S. aureus* cells, the peroxiredoxin AhpC was shown to enable the detoxification of low levels of 0.4 mM H_2_O_2_, which could originate from the aerobic respiration. Thus, our results support that catalases are scavengers of high mM levels of H_2_O_2_, whereas peroxiredoxins can only detoxify physiological µM H_2_O_2_ levels. However, the levels of H_2_O_2_ experienced by *S. aureus* during the oxidative burst were determined in the range of ~2 µM inside the phagosomes of neutrophils and macrophages [13,61,62]. In mammalian cells, the physiological intracellular H_2_O_2_ concentration was estimated in the range of 1–100 nM, while the extracellular H_2_O_2_ was 100-fold higher [63]. Thus, *S. aureus* might not experience such high doses of 10–100 mM H_2_O_2_ during interaction with immune cells. However, as a commensal bacterium, *S. aureus* colonizes the anterior nares and the nasopharynx together with competing microbes, such as *Streptococcus pneumoniae*, which generates millimolar levels of H_2_O_2_ by the lactate and pyruvate oxidases to kill competitive bacteria [64,65,66,67,68,69]. In a mouse infection model, KatA was required for nasal colonization of *S. aureus* [18]. Furthermore, the *katA* mutant was impaired in survival towards H_2_O_2_ produced by *S. pneumoniae* during nasal colonization [20,70]. Thus, microaerophilic H_2_O_2_ priming of *S. aureus* might provide an advantage in its ecological niche to resist the high H_2_O_2_ levels generated by competing microbes.

In contrast, KatA and the peroxiredoxins are not directly involved in the defence against HOCl stress in *S. aureus*. However, KatA might contribute to lowering external H_2_O_2_ and, subsequently, HOCl levels in the neutrophil phagosome. In support of this notion, KatA was found to be induced upon macrophage infection and to be essential for survival of *S. aureus* inside macrophages [21]. In addition, *S. aureus* strains with high catalase activity were more resistant to killing by neutrophils compared to strains with lower catalase activity [22]. These data support that KatA is an important defence mechanism in *S. aureus* against the respiratory burst of macrophages and neutrophils.

In addition, the peroxiredoxins AhpC, Tpx and Bcp were found to confer protection against CHP stress in *S. aureus* cells. Using Brx-roGFP2 biosensor measurements, the peroxiredoxin-deficient Δ*ahpC*, Δ*tpx* and Δ*bcp* mutants showed delayed regeneration of the reduced state of *E*_BSH_ after recovery from CHP stress. Thus, AhpC, Tpx and Bcp function in CHP detoxification and contribute to the maintenance of the cellular redox balance. The role of AhpC in CHP resistance has been previously demonstrated in *S. aureus* [20]. Similarly, the AhpC homologs of *B. subtilis*, *E. coli* and *S.* Typhimurium conferred resistance towards CHP stress [55,71]. In *E. coli*, the Δ*tpx* and Δ*bcp* mutants were more sensitive towards various OHPs, indicating that these peroxiredoxins are more specific to reduction of organic peroxide substrates [26,28]. Kinetic assays of the *E. coli* Tpx protein demonstrated the substrate specificity towards alkyl hydroperoxides over H_2_O_2_ [27]. Similarly, Bcp of *E. coli* has a fivefold higher V_max_/K_m_ value for linoleic acid hydroperoxide as a substrate compared to H_2_O_2_ [26].

## 5. Conclusions

Taken together, we have shown that the catalase KatA is the major player in aerobic H_2_O_2_ resistance in *S. aureus* and mediates priming to endogenous ROS levels generated during aerobic respiration to confer the constitutive H_2_O_2_ resistance towards the triggering stimulus in aerobic cells. In addition, KatA mediates microaerophilic priming by low H_2_O_2_ levels to prepare *S. aureus* cells for improved and adaptive resistance against otherwise lethal H_2_O_2_ doses. Furthermore, the peroxiredoxins AhpC, Tpx and Bcp were shown to contribute to CHP detoxification and resistance to ensure the survival and regeneration of the reduced *E*_BSH_ in *S. aureus* (Figure 8). In future studies, we aim to elucidate the functions of KatA and the peroxiredoxins in signal transduction, as redox-active chaperones and in cellular metabolism during aerobic growth and under oxidative stress in *S. aureus*.

## Data Availability

All reported data are available in the manuscript.

## Supplementary Materials

The following supporting information can be downloaded at: https://www.mdpi.com/article/10.3390/antiox11091793/s1: Figure S1: Complementation of KatA increases H_2_O_2_ resistance in *S. aureus*, but the catalase activity in the *katA* complemented strain is lower compared to the WT. Figure S2: Growth and catalase activity of the *ahpC* complemented strain. Figure S3: The Δ*katA*Δ*ahpC* double mutant is hypersensitive towards H_2_O_2_ and delayed in the recovery of reduced *E*_BSH_ after 0.4 mM H_2_O_2_. Figure S4: The *katA* complemented strain is not primable towards increased H_2_O_2_ resistance during microaerophilic growth. Figure S5: KatA and the peroxiredoxins AhpC, Tpx and Bcp are not involved in HOCl detoxification in *S. aureus*. Figure S6: The Northern blot images using the *katA* specific RNA probe show the *katA* transcripts (images of Figure 1H; two bioreplicates, three technical replicates). Figure S7: The Northern blot images using the *ahpC* specific RNA probe show the *ahpCF* operon transcripts (images of Figure 1H; two bioreplicates, three technical replicates). Figure S8: The Northern blot images using the *dps* specific RNA probe show the *dps* operon transcripts (images of Figure 1H; two bioreplicates, three technical replicates). Figure S9: The Northern blot images of priming experiments using the *katA* specific RNA probe show the *katA* transcripts (images of Figure 5B,C; three bioreplicates). Figure S10: The Northern blot images of priming experiments using the *ahpC* specific RNA probe show the *ahpCF* transcripts (images of Figure 5B,C; three bioreplicates). Figure S11: Native gels of catalase stains used for KatA activity assays (images of Figure 5F; two bioreplicates, two technical replicates). Figure S12: Native gels of catalase stains used for KatA activity assays (images of Figure S1D; three bioreplicates). Figure S13: Native gels of catalase stains used for KatA activity assays (images of Figure S2B; three bioreplicates). Table S1: Bacterial strains; Table S2: Plasmids; Table S3: Oligonucleotide primers. Table S4: CFU counts for the H_2_O_2_ survival assays. Table S5: CFU counts for microaerophilic H_2_O_2_ priming. Table S6: CFU counts for aerobic H_2_O_2_ priming. Table S7: CFU counts for the CHP survival assays. Table S8: CFU counts for HOCl survival assays.
