# Supplementary material for: The Catalase KatA Contributes to Microaerophilic H2O2 Priming to Acquire an Improved Oxidative Stress Resistance in Staphylococcus aureus
Source: Antioxidants (Basel). 2022 Sep 12;11(9):1793. doi: 10.3390/antiox11091793 (PMC9495333; doi:10.3390/antiox11091793)
Supplement: Supplementary file 1 [file antioxidants-11-01793-s001.zip › antioxidants-1878731-supplementary.pdf]

Table S1. Bacterial strains

Bacterial strains	Description	Reference
Escherichia coli		
DH5α	F-φ80dlacZ Δ(lacZYA-argF) U169 deoRsupE44ΔlacU169 (f80lacZDM15) hsdR17 recA1 endA1 (rk- mk+) supE44gyrA96 thi-1 gyrA69 relA1	[1]
Staphylococcus aureus		
RN4220	restriction negative strain/MSSA cloning intermediate derived from 8325-4	[2]
COL	Archaic HA-MRSA strain	[3]
COL-Δ*katA*	COL *katA* deletion mutant	[4]
COL-Δ*ahpC*	COL *ahpC* deletion mutant	This study
COL-Δ*bcp*	COL *bcp* deletion mutant	This study
COL-Δ*tpx*	COL *tpx* deletion mutant	This study
COL-Δ*perR*	COL *perR* deletion mutant	This study
COL-Δ*ahpC*Δ*katA*	COL *ahpC* and *katA* double deletion mutant	This study
COL pRB473-*brx-roGFP2*	COL WT expressing Brx-roGFP2 biosensor	[5]
COL-Δ*katA* pRB473-*brx-roGFP2*	COL *katA* deletion mutant expressing Brx-roGFP2	This study
COL-Δ*ahpC* pRB473-*brx-roGFP2*	COL *ahpC* deletion mutant expressing Brx-roGFP2	This study
COL-Δ*bcp* pRB473-*brx-roGFP2*	COL *bcp* deletion mutant expressing Brx-roGFP2	This study
COL-Δ*ahpC*Δ*katA*-pRB473-*brx-roGFP2*	COL *ahpC* and *katA* deletion mutant expressing Brx-roGFP2	This study
COL-Δ*tpx* pRB473-*brx-roGFP2*	COL *tpx* deletion mutant expressing Brx-roGFP2	This study
COL-Δ*katA::*pRB473-*katA*	COL *katA* mutant complemented with pRB473-*katA*	[4]
COL-Δ*ahpC::*pRB473-*ahpC-His*	COL *ahpC* mutant complemented with pRB473-*ahpC-His*	This study
COL-Δ*bcp*::pRB473-*bcp-His*	COL *bcp* mutant complemented with pRB473-*bcp-His*	This study
COL-Δ*tpx::*pRB473-*tpx-His*	COL *tpx* mutant complemented with pRB473-*tpx-His*	This study
Staphylococcus phage 81		[6]

Table S2. Plasmids

Plasmid	Description	Reference
pRB473-Xyl	pRB373-derivative, *E. coli/ S. aureus* shuttle vector, containing xylose-inducible P_{Xyl} promoter, Amp^r, Cm^r	[7, 8]
pRB473-Xyl-*brx-roGFP2*	pRB473-derivative expressing *brx-roGFP2* under P_{Xyl}	[5]
pRB473-Xyl-*katA*	pRB473-derivative expressing *katA* under P_{Xyl}	[4]
pRB473-Xyl-*ahpC-His*	pRB473-derivative expressing His-tagged *ahpC* under P_{Xyl}	This study
pRB473-Xyl-*bcp-His*	pRB473-derivative expressing His-tagged *bcp* under P_{Xyl}	This study
pRB473-Xyl-*tpx-His*	pRB473-derivative expressing His-tagged *tpx* under P_{Xyl}	This study

Table S3. Oligonucleotide primers

Primer name	Sequence (5' to 3')
pMAD-ahpC-for-BglII	CGCAGATCTCCACTCCTCGATACTTTACAAT
pMAD-ahpC-f1-rev	CTACTAAATCTAAACCAGGTTGCTGTAAATGGTAAGATTTCTTTG
pMAD-ahpC-f2-for	CAAAGAAATCTTACCATTTACAGCAACCTGGTTTAGATTTAGTAG
pMAD-ahpC-rev-SalI	CCAGTCGACCATCAATCATAGAATGCGTGAT
pMAD-bcp-for-BglII	CGCAGATCTCTTTTAGTATATGCACGTGCAA
pMAD-bcp-f1-rev	TGTTTTTAAGTTCTTCTATTTGTGTGGAAATTGTTCTCCTTTTTGC
pMAD-bcp-f2-for	GCAAAAAGGAGAACAATTTCCACACAAATAGAAGAACTTAAAAACA
pMAD-bcp-rev-SalI	CCAGTCGACGCTAACTTCGCAGTTCTAGTA
pMAD-tpx-for-BglII	CGCAGATCTACGCACGTTTACTCAATTTACA
pMAD-tpx-f1-rev	TTAAATATTTTTGTATGCAGCTAAACCTTTGAATGTTATTTCAGTCAT
pMAD-tpx-f2-for	ATGACTGAAATAACATTCAAAGGTTTAGCTGCATACAAAAATATTTAA
pMAD-tpx-rev-SalI	CCAGTCGACCAGGTAACACTTCTTTTACAGT
pMAD-perR-for-BglII	CGCAGATCTAATCACTTGAAAGCACATTACCA
pMAD-perR-f1-rev	TCTTGGCATTCTTTACAAACTCATTGATTCTATTTCAACACTCAT
pMAD-perR-f2-for	ATGAGTGTTGAAATAGAATCAATGAGTTTGTAAAGAATGCCAAGA
pMAD-perR-rev-SalI	CCAGTCGACGAATTTCAATAGTCAAATTTACAC
pRB-ahpC-for-BamHI-	TAGGGATCCATTCTTAGGAGGAAGATATTTATG
pRB-ahpC-rev-KpnI-His	CTCGGTACCTTAGTGATGGTGATGGTGATGGATTTTACCTACTAAATCTAAA
pRB-bcp-for-BamHI	TAGGGATCCAATGAAGAAAAAGGTGATTATATG
pRB-bcp-rev-KpnI-His	CTCGGTACCTCAGTGATGGTGATGGTGATGCCCCAAAATGTTTTTAAGTTCTT
pRB-tpx-for-BamHI	TAGGGATCCATGCAGGAGGTCAAGTATATGACTGAAATAACATTCAAAG
pRB-tpx-rev-SacI-His	CTCGAGCTCTTAGTGATGGTGATGGTGATGAATATTTTTGTATGCAGC
katA-NB-for	AAAGGTTCTGGTGCATTTGG
katA-NB-rev	CTAATACGACTCACTATAGGGAGAAATGTGTTCCTCCACCTTGG
ahpC-NB-for	TCCTGCTGACTTCTCATTCGT
ahpC-NB-rev	CTAATACGACTCACTATAGGGAGAGGTTGCAATGTTTTAGCGCC

Restriction sites are underlined.

Supplementary References

[1] Studier, FW, Moffatt, BA. Use of bacteriophage-T7 RNA-polymerase to direct selective high-level expression of cloned genes. *J Mol Biol 189*(1): 113-130, 1986.

[2] Kreiswirth, BN, Lofdahl, S, Betley, MJ, O'reilly, M, Schlievert, PM, Bergdoll, MS, Novick, RP. The toxic shock syndrome exotoxin structural gene is not detectably transmitted by a prophage. *Nature 305*(5936): 709-712, 1983.

[3] Shafer, WM, Iandolo, JJ. Genetics of staphylococcal enterotoxin B in methicillin-resistant isolates of *Staphylococcus aureus*. *Infect. Immun. 25*(3): 902-911, 1979.

[4] Linzner, N, Fritsch, VN, Busche, T, Tung, QN, Loi, VV, Bernhardt, J, Kalinowski, J, Antelmann, H. The plant-derived naphthoquinone lapachol causes an oxidative stress response in *Staphylococcus aureus*. *Free Radic. Biol. Med. 158*126-136, 2020.

[5] Loi, VV, Harms, M, Müller, M, Huyen, NTT, Hamilton, CJ, Hochgräfe, F, Pane-Farre, J, Antelmann, H. Real-time imaging of the bacillithiol redox potential in the human pathogen *Staphylococcus aureus* using a genetically encoded bacilliredoxin-fused redox biosensor. *Antioxid. Redox Signal. 26*(15): 835-848, 2017.

[6] Rosenblum, ED, Tyrone, S. Serology, density, and morphology of staphylococcal phages. *J Bacteriol 88*(6): 1737-1742, 1964.

[7] Brückner, R, Wagner, E, Götz, F. Characterization of a sucrase gene from *Staphylococcus xylosus*. *J. Bacteriol. 175*(3): 851-857, 1993.

[8] Pöther, DC, Gierok, P, Harms, M, Mostertz, J, Hochgräfe, F, Antelmann, H, Hamilton, CJ, Borovok, I, Lalk, M, Aharonowitz, Y, Hecker, M. Distribution and infection-related functions of bacillithiol in *Staphylococcus aureus*. *Int. J. Med. Microbiol. 303*(3): 114-123, 2013.

Table S4. CFU counts for the H_2O_2 survival assays

A) Average CFU/ml of *S. aureus* COL WT, Δ*katA*, Δ*ahpC*, Δ*tpx* and Δ*bcp* mutants and the *ahpC* complemented strain before (0 h) and after 2 and 4 h of exposure to 40 mM H_2O_2
(The % survival rates are shown in **Fig. 1E**)

	CFU/ml		
**Strains**	**0 h**	**2 h**	**4 h**
WT	$6.63 \times 10^7 \pm 1.11 \times 10^7$	$2.12 \times 10^7 \pm 1.75 \times 10^7$	$5.28 \times 10^6 \pm 8.07 \times 10^5$
Δ*katA*	$8.90 \times 10^7 \pm 2.62 \times 10^7$	0	0
Δ*ahpC*	$7.33 \times 10^7 \pm 1.59 \times 10^7$	$2.37 \times 10^8 \pm 7.51 \times 10^7$	$4.87 \times 10^8 \pm 4.53 \times 10^7$
ahpC	$1.24 \times 10^8 \pm 4.76 \times 10^7$	$2.89 \times 10^8 \pm 3.76 \times 10^7$	$3.59 \times 10^8 \pm 1.28 \times 10^8$
Δ*tpx*	$2.82 \times 10^7 \pm 2.50 \times 10^7$	$2.44 \times 10^6 \pm 1.20 \times 10^6$	$1.65 \times 10^6 \pm 5.51 \times 10^5$
Δ*bcp*	$9.57 \times 10^7 \pm 3.57 \times 10^7$	$3.29 \times 10^7 \pm 4.86 \times 10^6$	$1.04 \times 10^7 \pm 2.09 \times 10^6$

B) Average CFU/ml of *S. aureus* COL WT, the Δ*katA* and Δ*ahpC*Δ*katA* mutants and the *katA* complemented strain before (0 h) and after 2 and 4 h of exposure to 1 mM H_2O_2.
(The % survival rates are shown in **Fig. 1F**)

	CFU/ml		
**Strains**	**0 h**	**2 h**	**4 h**
WT	$6.93 \times 10^7 \pm 3.23 \times 10^7$	$2.17 \times 10^8 \pm 7.46 \times 10^7$	$4.90 \times 10^8 \pm 1.98 \times 10^8$
Δ*katA*	$8.00 \times 10^7 \pm 3.71 \times 10^7$	$2.35 \times 10^7 \pm 7.41 \times 10^6$	$3.41 \times 10^6 \pm 1.81 \times 10^6$
katA	$6.30 \times 10^7 \pm 1.36 \times 10^7$	$8.43 \times 10^7 \pm 1.09 \times 10^7$	$2.46 \times 10^8 \pm 3.99 \times 10^7$
Δ*ahpC*Δ*katA*	$8.87 \times 10^7 \pm 2.71 \times 10^7$	$9.33 \times 10^6 \pm 1.41 \times 10^6$	$3.50 \times 10^5 \pm 3.89 \times 10^5$

C) Average CFU/ml of log and stationary phase cells of *S. aureus* COL WT and the Δ*katA* mutant before (0 h) and after 1 and 2 h of exposure to 40 mM H_2O_2.
(The % survival rates are shown in **Fig. 1G)**

	CFU/ml		
**Strains**	**0 h**	**1 h**	**2 h**
WT log	$8.93 \times 10^7 \pm 1.16 \times 10^7$	$5.97 \times 10^7 \pm 1.30 \times 10^7$	-
	$6.63 \times 10^7 \pm 1.11 \times 10^7$	-	$2.12 \times 10^7 \pm 1.75 \times 10^7$
WT stat	$1.31 \times 10^9 \pm 3.21 \times 10^8$	$1.40 \times 10^9 \pm 3.36 \times 10^8$	$1.74 \times 10^9 \pm 2.93 \times 10^8$
Δ*katA* log	$8.90 \times 10^7 \pm 2.62 \times 10^7$	0	0
Δ*katA* stat	$1.13 \times 10^9 \pm 9.28 \times 10^7$	$4.03 \times 10^2 \pm 6.35 \times 10^2$	0

Table S5. CFU counts for microaerophilic H_2O_2 priming

A) Average CFU/ml of the naïve (C), primed (P), primed and triggered (PT) and triggered (T) *S. aureus* COL WT and the Δ*katA* mutant during the microaerophilic growth before (0 h) and after 2 and 4 h of exposure to 10 mM H_2O_2. (The % survival rates are shown in **Fig. 3 D, E**)

	**CFU/ml**		
**WT**	**0 h**	**2 h**	**4 h**
C	$2.01 \times 10^8 \pm 2.07 \times 10^7$	$2.32 \times 10^8 \pm 8.99 \times 10^6$	$2.29 \times 10^8 \pm 2.06 \times 10^7$
P	$1.93 \times 10^8 \pm 1.79 \times 10^7$	$2.25 \times 10^8 \pm 4.33 \times 10^7$	$2.95 \times 10^8 \pm 1.46 \times 10^7$
PT	$2.39 \times 10^8 \pm 2.57 \times 10^7$	$1.25 \times 10^8 \pm 8.52 \times 10^6$	$1.70 \times 10^8 \pm 3.35 \times 10^7$
T	$2.16 \times 10^8 \pm 1.32 \times 10^7$	$1.44 \times 10^5 \pm 5.00 \times 10^4$	$1.35 \times 10^4 \pm 3.02 \times 10^3$
**Δ*katA***	**0 h**	**2 h**	**4 h**
C	$2.22 \times 10^8 \pm 1.33 \times 10^7$	$2.53 \times 10^8 \pm 4.32 \times 10^7$	$2.42 \times 10^8 \pm 4.23 \times 10^7$
P	$7.87 \times 10^7 \pm 2.51 \times 10^7$	$1.12 \times 10^8 \pm 3.34 \times 10^7$	$8.93 \times 10^7 \pm 1.61 \times 10^7$
PT	$5.64 \times 10^7 \pm 1.98 \times 10^7$	$7.50 \times 10^0 \pm 8.29 \times 10^0$	0
T	$2.50 \times 10^8 \pm 1.98 \times 10^7$	$1.50 \times 10^1 \pm 2.06 \times 10^1$	0

B) Average CFU/ml of the naïve (C), primed (P), primed and triggered (PT) and triggered (T) *S. aureus* COL *katA* complemented strain during the microaerophilic growth under before (0 h) and after 2 and 4 h of exposure to 10 mM H_2O_2.
(The % survival rates are shown in **Fig. S4C**)

	**CFU/ml**		
katA	**0 h**	**2 h**	**4 h**
C	$1.58 \times 10^8 \pm 3.06 \times 10^7$	$1.57 \times 10^8 \pm 6.26 \times 10^6$	$1.71 \times 10^8 \pm 3.52 \times 10^7$
P	$1.63 \times 10^8 \pm 2.12 \times 10^7$	$1.55 \times 10^8 \pm 2.35 \times 10^7$	$1.92 \times 10^8 \pm 4.22 \times 10^7$
PT	$1.70 \times 10^8 \pm 2.27 \times 10^7$	$4.91 \times 10^3 \pm 3.12 \times 10^3$	$4.24 \times 10^2 \pm 2.87 \times 10^2$
T	$1.59 \times 10^8 \pm 2.90 \times 10^7$	$1.75 \times 10^3 \pm 9.17 \times 10^2$	$4.20 \times 10^2 \pm 3.35 \times 10^2$

Table S6. CFU counts for aerobic H_2O_2 priming

A) Average CFU/ml of the naïve (C), primed (P), primed and triggered (PT) and triggered (T) *S. aureus* COL WT during the aerobic growth before (0 h) and after 2 and 4 h of exposure to 10 mM H_2O_2. (The % survival rates are shown in **Fig. 4C**)

	**CFU/ml**		
**WT**	**0 h**	**2 h**	**4 h**
C	$6.01 \times 10^7 \pm 6.12 \times 10^6$	$3.45 \times 10^8 \pm 1.83 \times 10^7$	$1.04 \times 10^9 \pm 2.98 \times 10^8$
P	$5.31 \times 10^7 \pm 1.49 \times 10^7$	$3.02 \times 10^8 \pm 7.17 \times 10^7$	$1.01 \times 10^9 \pm 3.15 \times 10^8$
PT	$5.31 \times 10^7 \pm 1.49 \times 10^7$	$4.32 \times 10^7 \pm 2.23 \times 10^7$	$1.03 \times 10^8 \pm 4.44 \times 10^7$
T	$6.01 \times 10^7 \pm 6.12 \times 10^6$	$3.60 \times 10^7 \pm 1.34 \times 10^7$	$7.68 \times 10^7 \pm 4.19 \times 10^7$

B) Average CFU/ml of the naïve (C), primed (P), primed and triggered (PT) and triggered (T) *S. aureus* COL WT during the aerobic growth before (0 h) and after 2 and 4 h of exposure to 40 mM H_2O_2. (The % survival rates are shown in **Fig. 4F**)

	**CFU/ml**		
**WT**	**0 h**	**2 h**	**4 h**
C	$6.08 \times 10^7 \pm 4.01 \times 10^6$	$4.46 \times 10^8 \pm 1.45 \times 10^8$	$9.17 \times 10^8 \pm 2.08 \times 10^8$
P	$5.44 \times 10^7 \pm 9.86 \times 10^6$	$2.03 \times 10^8 \pm 3.29 \times 10^7$	$5.35 \times 10^8 \pm 1.21 \times 10^8$
PT	$5.44 \times 10^7 \pm 9.86 \times 10^6$	$4.58 \times 10^6 \pm 1.93 \times 10^6$	$5.74 \times 10^5 \pm 5.09 \times 10^5$
T	$6.08 \times 10^7 \pm 4.01 \times 10^6$	$6.99 \times 10^6 \pm 2.95 \times 10^6$	$3.18 \times 10^6 \pm 1.01 \times 10^6$

Table S7. CFU counts for the CHP survival assays

Average CFU/ml of the *S. aureus* COL WT, the Δ*katA*, Δ*ahpC*, Δ*tpx* and Δ*bcp* mutants and complemented strains before (0 h) and after 2 and 4 h of exposure to 40 mM H_2O_2.

(The % survival rates are shown in **Fig. 6G, H**)

	CFU/ml		
**Strains**	**0 h**	**2 h**	**4 h**
COL WT	$6.70 \times 10^7 \pm 1.98 \times 10^7$	$2.46 \times 10^7 \pm 1.12 \times 10^7$	$2.95 \times 10^7 \pm 1.54 \times 10^7$
Δ*katA*	$1.23 \times 10^8 \pm 5.19 \times 10^7$	$1.48 \times 10^8 \pm 2.52 \times 10^7$	$1.65 \times 10^8 \pm 8.26 \times 10^7$
Δ*ahpC*	$6.87 \times 10^7 \pm 1.92 \times 10^7$	$2.37 \times 10^7 \pm 6.87 \times 10^6$	$5.95 \times 10^6 \pm 3.93 \times 10^6$
Δ*tpx*	$2.30 \times 10^7 \pm 2.32 \times 10^7$	$5.30 \times 10^6 \pm 3.70 \times 10^6$	$2.31 \times 10^6 \pm 1.77 \times 10^6$
Δ*bcp*	$7.37 \times 10^7 \pm 1.14 \times 10^7$	$1.72 \times 10^7 \pm 6.26 \times 10^6$	$7.62 \times 10^6 \pm 6.08 \times 10^6$
katA	$9.83 \times 10^7 \pm 6.04 \times 10^7$	$9.46 \times 10^7 \pm 5.27 \times 10^7$	$8.77 \times 10^7 \pm 4.71 \times 10^7$
ahpC	$1.32 \times 10^8 \pm 1.47 \times 10^7$	$6.97 \times 10^7 \pm 4.60 \times 10^7$	$4.40 \times 10^7 \pm 1.27 \times 10^7$
tpx	$9.65 \times 10^7 \pm 2.35 \times 10^7$	$3.42 \times 10^7 \pm 2.05 \times 10^7$	$4.23 \times 10^7 \pm 2.04 \times 10^7$
bcp	$1.14 \times 10^8 \pm 2.95 \times 10^7$	$4.22 \times 10^7 \pm 1.73 \times 10^7$	$3.71 \times 10^7 \pm 8.71 \times 10^6$

Table S8. CFU counts for HOCl survival assays

Average CFUs/ml of the *S. aureus* COL WT and Δ*katA*, Δ*ahpC*, Δ*tpx* and Δ*bcp* mutant strains before (0 h) and after 2 and 4 h of exposure to 2.5 mM HOCl.

(The % survival rates are shown in **Fig. S5E**)

	CFU/ml		
**Strains**	**0 h**	**2 h**	**4 h**
COL WT	$7.84 \times 10^7 \pm 1.39 \times 10^7$	$3.93 \times 10^7 \pm 1.05 \times 10^7$	$1.26 \times 10^7 \pm 2.69 \times 10^6$
Δ*katA*	$1.14 \times 10^8 \pm 5.24 \times 10^7$	$5.75 \times 10^7 \pm 3.78 \times 10^7$	$1.92 \times 10^7 \pm 1.36 \times 10^7$
Δ*ahpC*	$7.23 \times 10^7 \pm 1.45 \times 10^7$	$2.71 \times 10^7 \pm 5.98 \times 10^6$	$1.45 \times 10^7 \pm 1.54 \times 10^6$
Δ*tpx*	$3.41 \times 10^7 \pm 2.52 \times 10^7$	$1.47 \times 10^7 \pm 1.05 \times 10^7$	$6.94 \times 10^6 \pm 8.04 \times 10^6$
Δ*bcp*	$1.00 \times 10^8 \pm 2.76 \times 10^7$	$3.75 \times 10^7 \pm 1.49 \times 10^7$	$2.70 \times 10^7 \pm 1.08 \times 10^7$

Fig. S1

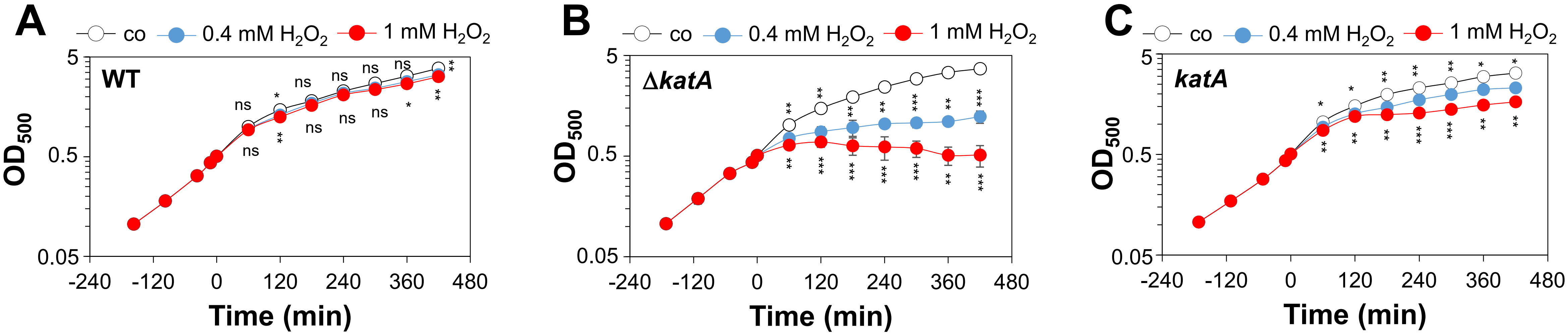

A:

Time (h)	1	2	3	4	5	6	7
Control vs 0.4 mM H_2O_2	ns	*	ns	ns	ns	ns	**
Control vs 1.0 mM H_2O_2	ns	**	ns	ns	ns	*	**

B:

Time (h)	1	2	3	4	5	6	7
Control vs 0.4 mM H_2O_2	**	**	**	**	***	**	***
Control vs 1.0 mM H_2O_2	**	***	***	***	***	**	***

C:

Time (h)	1	2	3	4	5	6	7
Control vs 0.4 mM H_2O_2	*	*	**	**	**	*	*
Control vs 1.0 mM H_2O_2	**	**	**	***	***	**	**

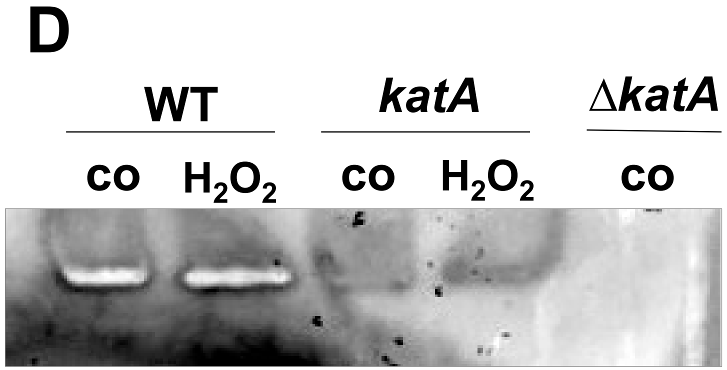

Fig. S1. Complementation of KatA increases the H_2O_2 resistance in *S. aureus* (A-C), but the catalase activity in the *katA* complemented strain is lower compared to the WT (D). Growth curves of the *S. aureus* COL WT **(A)**, the Δ*katA* mutant **(B)** and the *katA* complemented strain **(C)** after exposure to 0.4 and 1 mM H_2O_2 stress at an OD_{500} of 0.5. Mean values and SD of four biological replicates are presented. **(D)** Catalase staining of aerobically grown *S. aureus* COL WT, the Δ*katA* mutant and the *katA* complemented strain before (co) and after exposure to 100 µM H_2O_2 at an OD_{500} of 0.5. Protein extracts were separated by native PAGE and the catalase activity was determined using diamidobenzidine staining as described in the Methods. The statistics is shown below the graphs and was calculated using the Student's unpaired two-tailed t-test by the graph prism software for co vs. 0.4 mM and co vs. 1 mM H_2O_2. Symbols are $^{ns}p > 0.05$, $*p \le 0.05$,$**p \le 0.01$ and $***p \le 0.001$.

Fig. S2

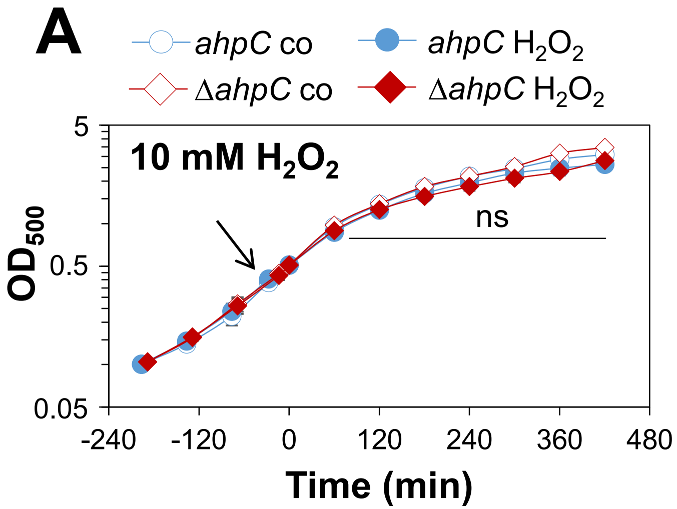

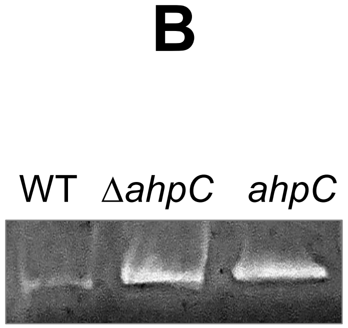

Fig. S2. Growth and catalase activity of the *ahpC* complemented strain. (A) Growth curves of *S. aureus* COL Δ*ahpC* mutant and *ahpC* complemented strains in RPMI medium before (co) and after exposure to 10 mM H_2O_2 **(A)**. Mean values and SD of 4 biological replicates are presented. The statistics was determined using a Student's unpaired two-tailed t-test by graph prism. Symbols are: $^{ns}p > 0.05$ **(B)** Catalase staining shows higher catalase activity in the Δ*ahpC* mutant and *ahpC* complemented strain compared to the WT at control conditions.

Fig. S3

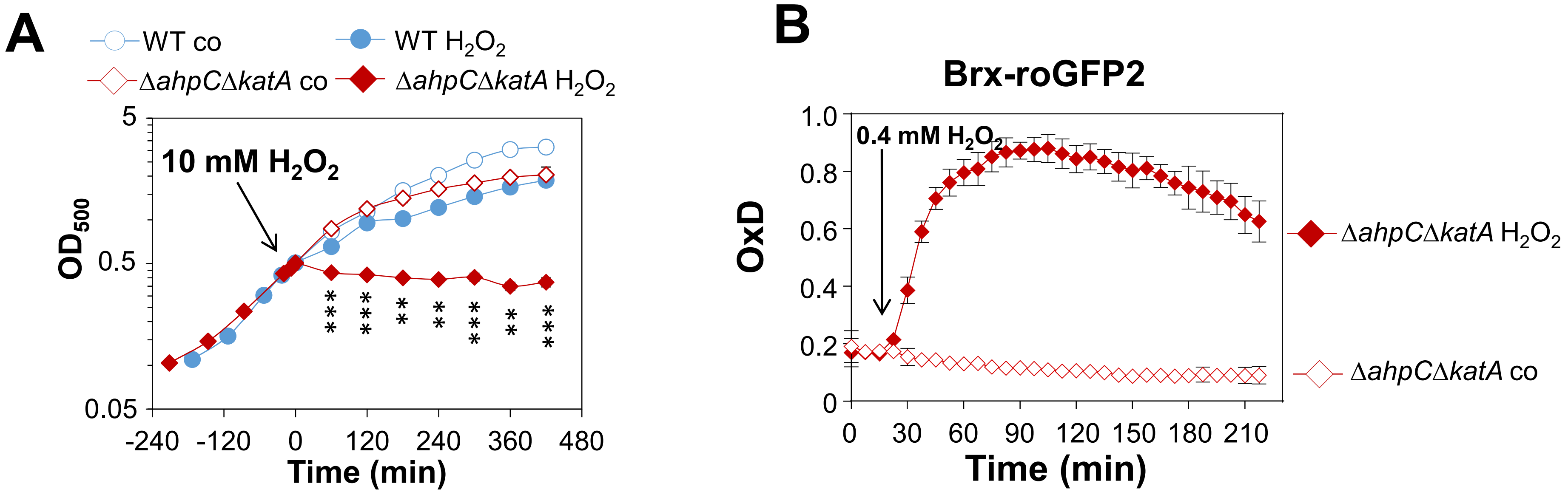

Fig. S3. The Δ*katA ahpC* double mutant is hypersensitive towards H_2O_2 and delayed in recovery of the E_{BSH} after 0.4 mM H_2O_2 (A) Growth curves of *S. aureus* COL WT and the Δ*ahpC*Δ*katA* double mutant in RPMI medium before (co) and after exposure to 10 mM H_2O_2 at an OD_{500} of 0.5. The survival rates were calculated relative to the untreated control, which was set to 100%. Mean values and SD of 3-4 biological replicates are shown. The statistics was calculated using the Student's unpaired two-tailed t-test by the graph prism software. Symbols are $^*p \leq 0.05$, $^{**}p \leq 0.01$ and $^{***}p \leq 0.001$. **(B)** The Brx-roGFP2 biosensor in the *S. aureus* COL Δ*ahpC*Δ*katA* double mutant showed a fast oxidation and a delay in recovery of the reduced E_{BSH} after 0.4 mM H_2O_2. The oxidation degree (OxD) of the Brx-roGFP2 response was calculated based on the 405/488 nm excitation ratios and normalized to fully reduced and oxidized controls. Mean values and SD of the OxD values are presented from 3 biological replicates.

Fig. S4

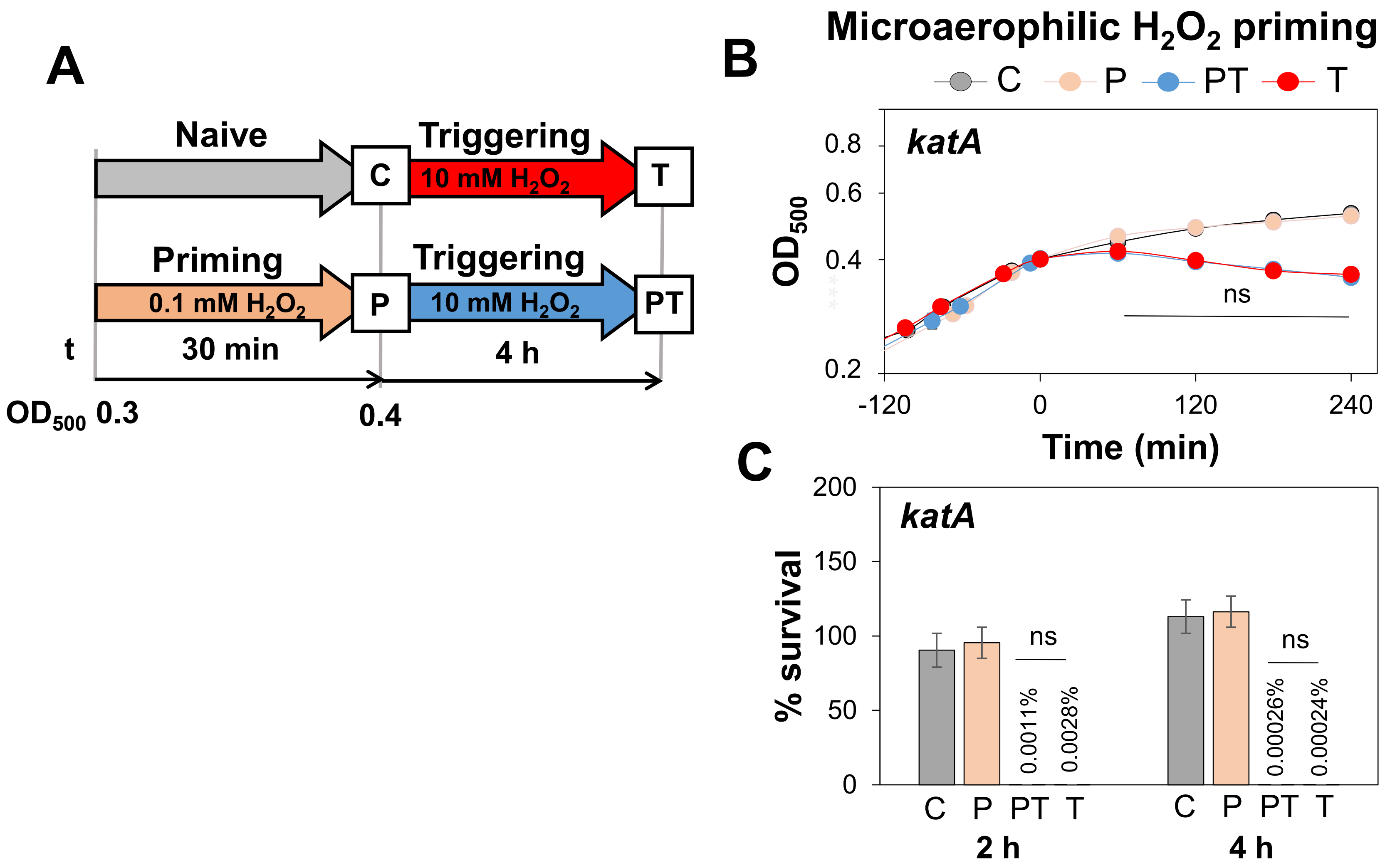

Fig. S4. The *katA* complemented strain is not primable towards increased H_2O_2 resistance during the microaerophilic growth. (A) Setup for microaerophilic priming and triggering experiments. The *S. aureus katA* complemented strain was primed during the log phase with 0.1 mM H_2O_2 for ~30 min **(P)** and subsequently treated with 10 mM H_2O_2 as triggering stimulus **(PT).** The growth curves **(B)** and survival rates **(C)** were determined in naïve (C), primed (P), primed and triggered (PT) and triggered cells (T). The survival rates were calculated after 2 and 4 h of H_2O_2 stress relative to untreated control cells. The results are from 3-4 biological replicates. Error bars represent the SD. The statistics was determined using a Student's unpaired two-tailed t-test by graph prism. Symbols are: $^{ns}p > 0.05$.

Fig. S5

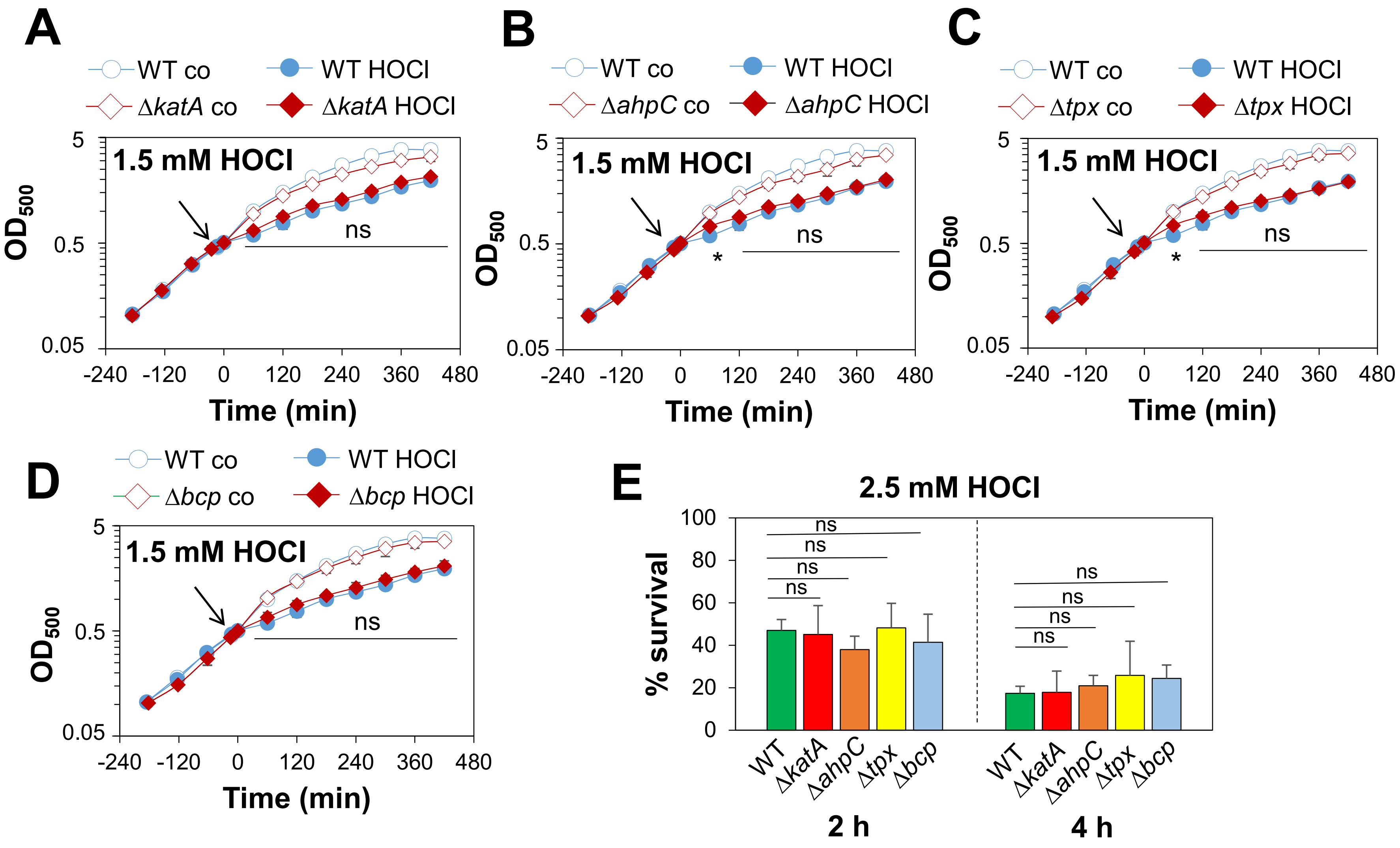

Fig. S5. KatA and the peroxiredoxins AhpC, Tpx and Bcp are not involved in HOCl detoxification in *S. aureus*. (A-C) Growth curves of *S. aureus* COL WT, Δ*katA* **(A)**, Δ*ahpC* **(B)**, Δ*tpx* **(C)** and Δ*bcp* mutants **(D)** in RPMI medium before (co) and after exposure to 1.5 mM HOCl stress during the log phase. **(D)** Survival rates were determined as CFUs of *S. aureus* COL WT, Δ*katA,* Δ*ahpC,* Δ*tpx* and Δ*bcp* mutants at 2 and 4 h after treatment with 2.5 mM HOCl. Survival of the untreated control was set to 100%. Mean values and SD of 4-5 biological replicates are presented. The statistics was determined using a Student's unpaired two-tailed t-test by graph prism. Symbols are: $^{ns}p > 0.05$, $^{*}p \leq 0.05$

Fig. S6

A

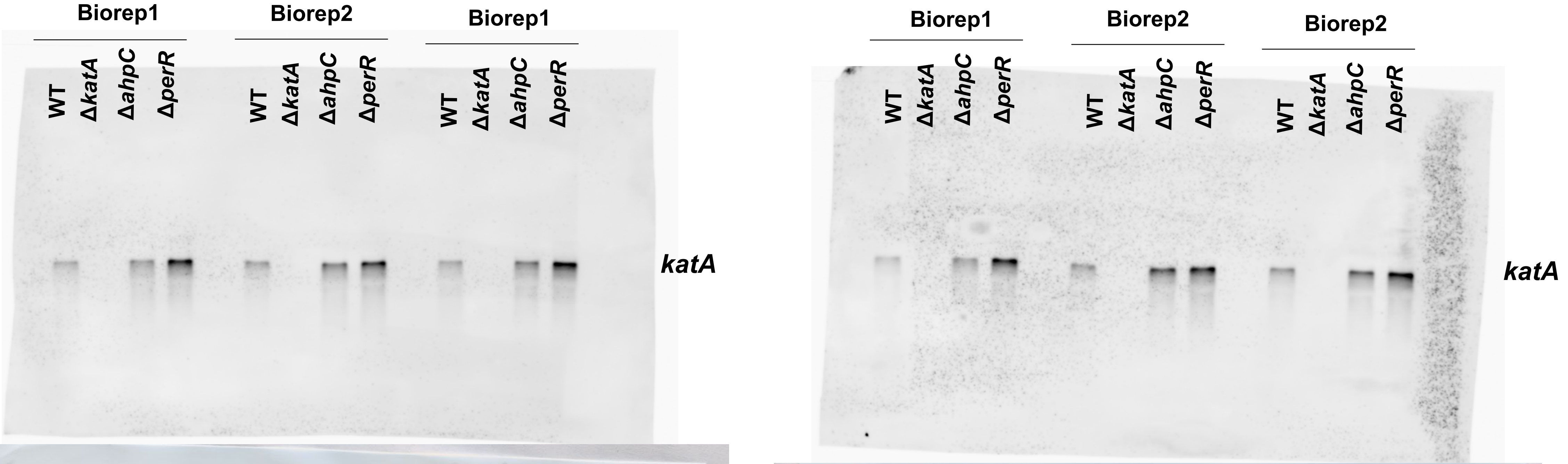

B

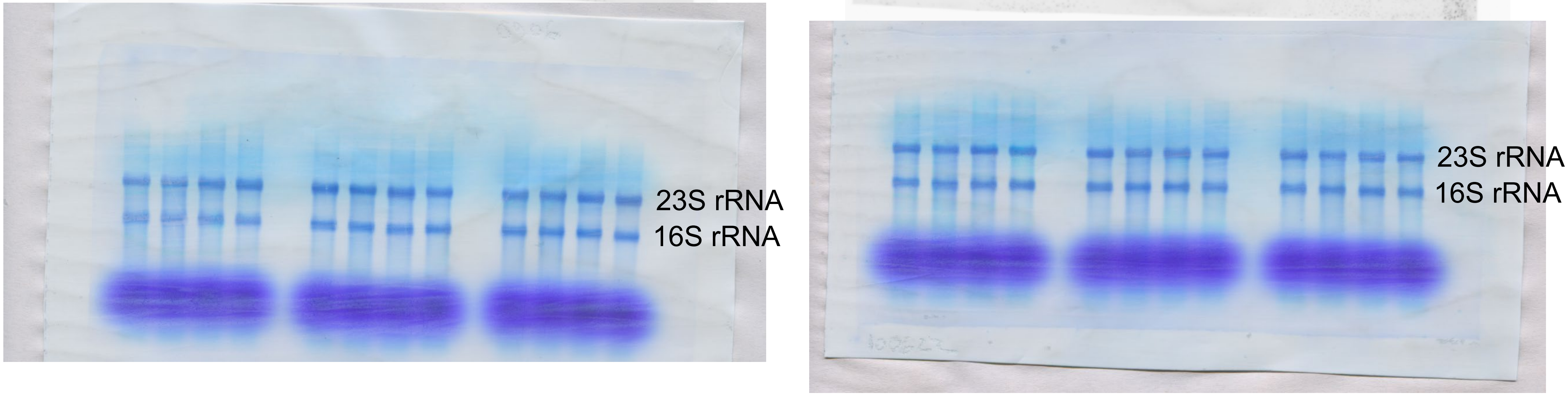

Fig. S6. A) The Northern blot images using the *katA* specific RNA probe show the *katA* transcripts (images of Fig. 1H, 2 bioreplicates, 3 technical replicates). **B)** The methylen blue stain of the Northern blots used as loading control for total RNA (images of Fig. 1H, 2 bioreplicates, 3 technical replicates). Labelling of RNA samples is as above for the detection of the *katA* transcript. The bands for the 16S and 23S rRNAs are indicated.

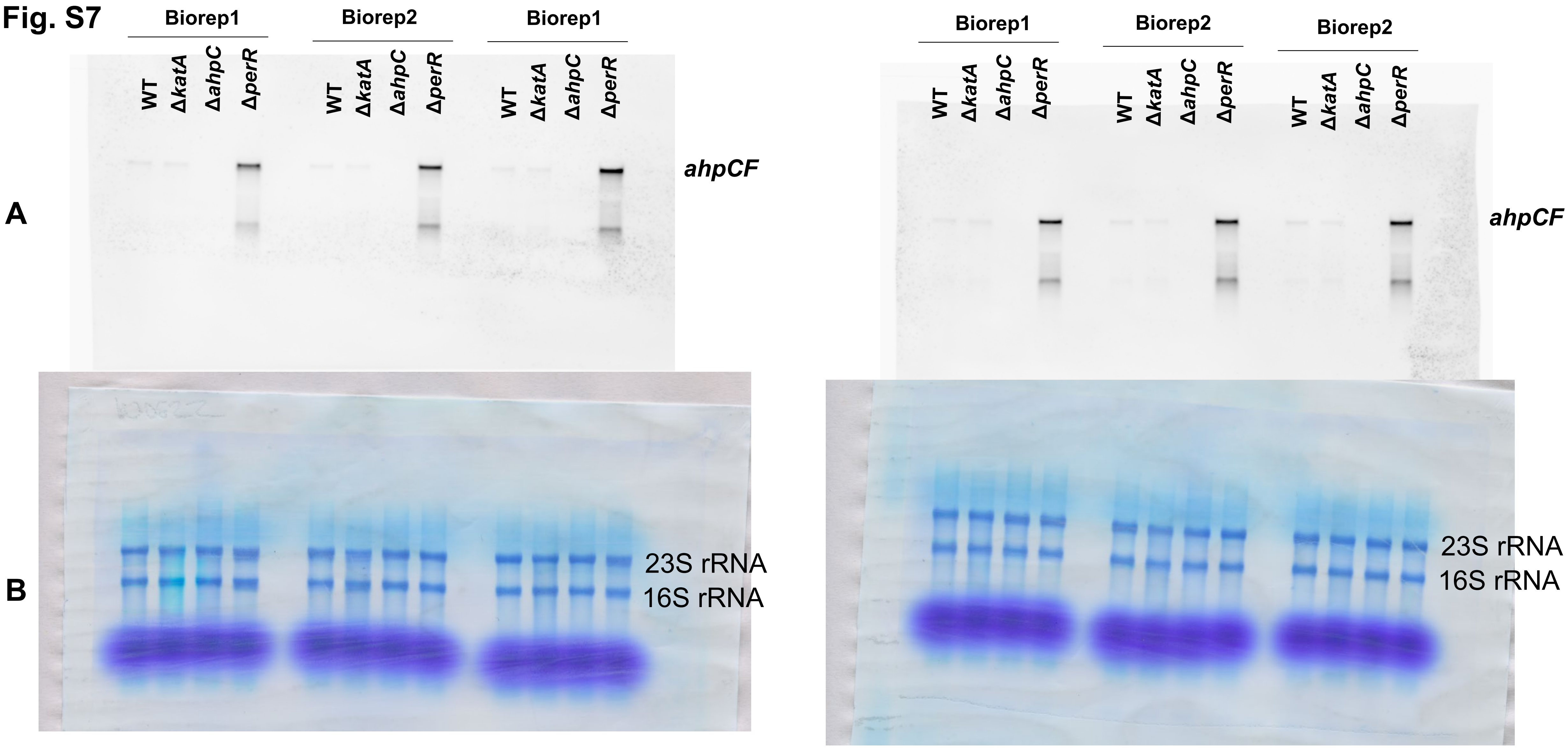

Fig. S7. A) The Northern blot images using the *ahpC* specific RNA probe show the *ahpCF* operon transcripts (images of Fig. 1H, 2 bioreplicates, 3 technical replicates). **B)** The methylen blue stain of the Northern blots used as loading control for total RNA (images of Fig. 1H, 2 bioreplicates, 3 technical replicates). Labelling of RNA samples is as above for the detection of the *ahpCF* transcript. The bands for the 16S and 23S rRNAs are indicated.

Fig. S8

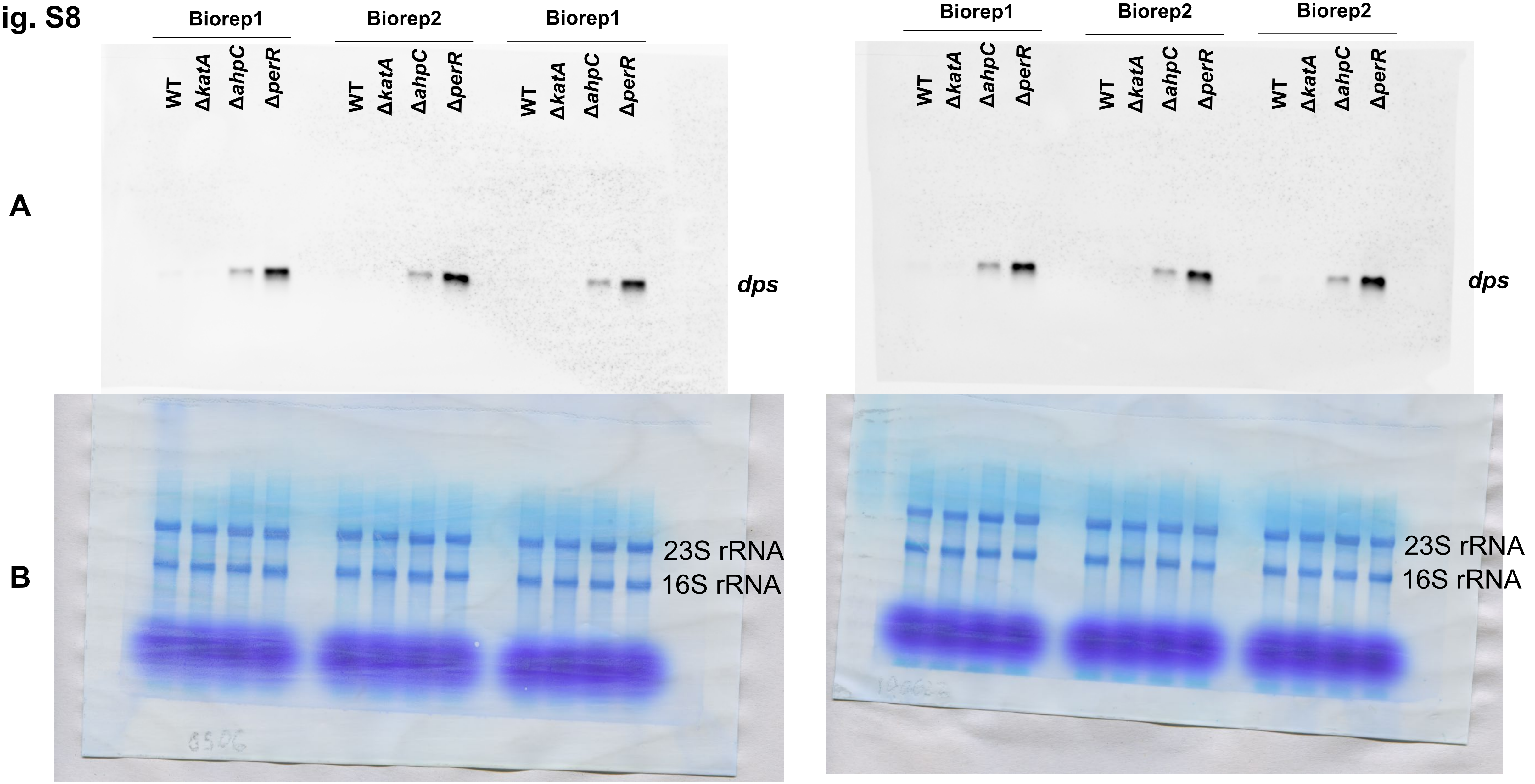

Fig. S8. A) The Northern blot images using the *dps* specific RNA probe show the *dps* transcripts (images of Fig. 1H, 2 bioreplicates, 3 technical replicates). **B)** The methylen blue stain of the Northern blots used as loading control for total RNA (images of Fig. 1H, 2 bioreplicates, 3 technical replicates). Labelling of RNA samples is as above for the detection of the *dps* transcript. The bands for the 16S and 23S rRNAs are indicated.

Fig. S9

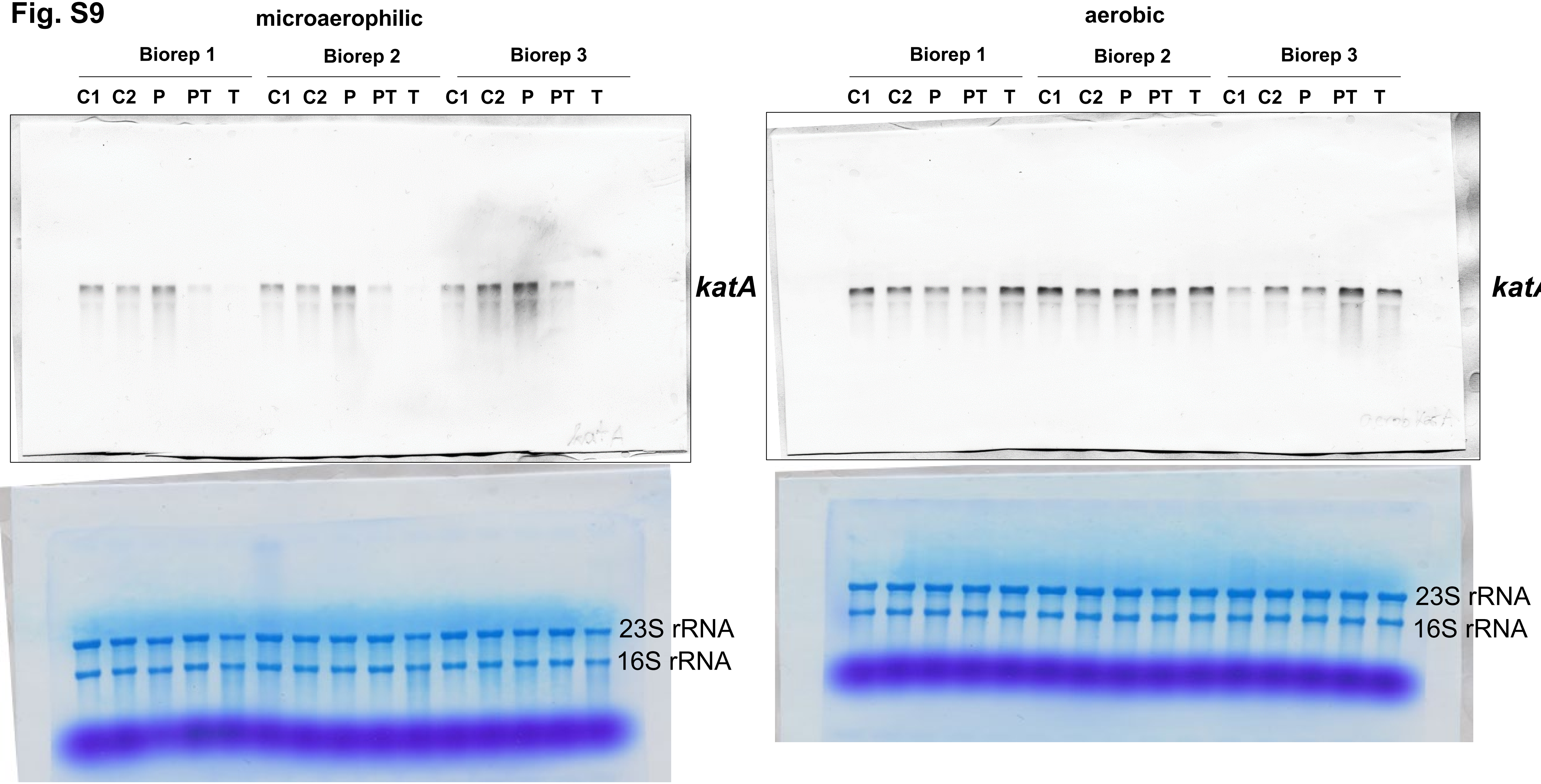

Fig. S9. A) The Northern blot images of priming experiments using the *katA* specific RNA probe show the *katA* transcripts (images of Fig. 5B,C, 3 bioreplicates). **B)** The methylen blue stain of the Northern blots used as loading control for total RNA (images of Fig. 5B,C; 3 bioreplicates). Labelling of RNA samples is as above for the detection of the *katA* transcript. The bands for the 16S and 23S rRNAs are indicated.

Fig. S10

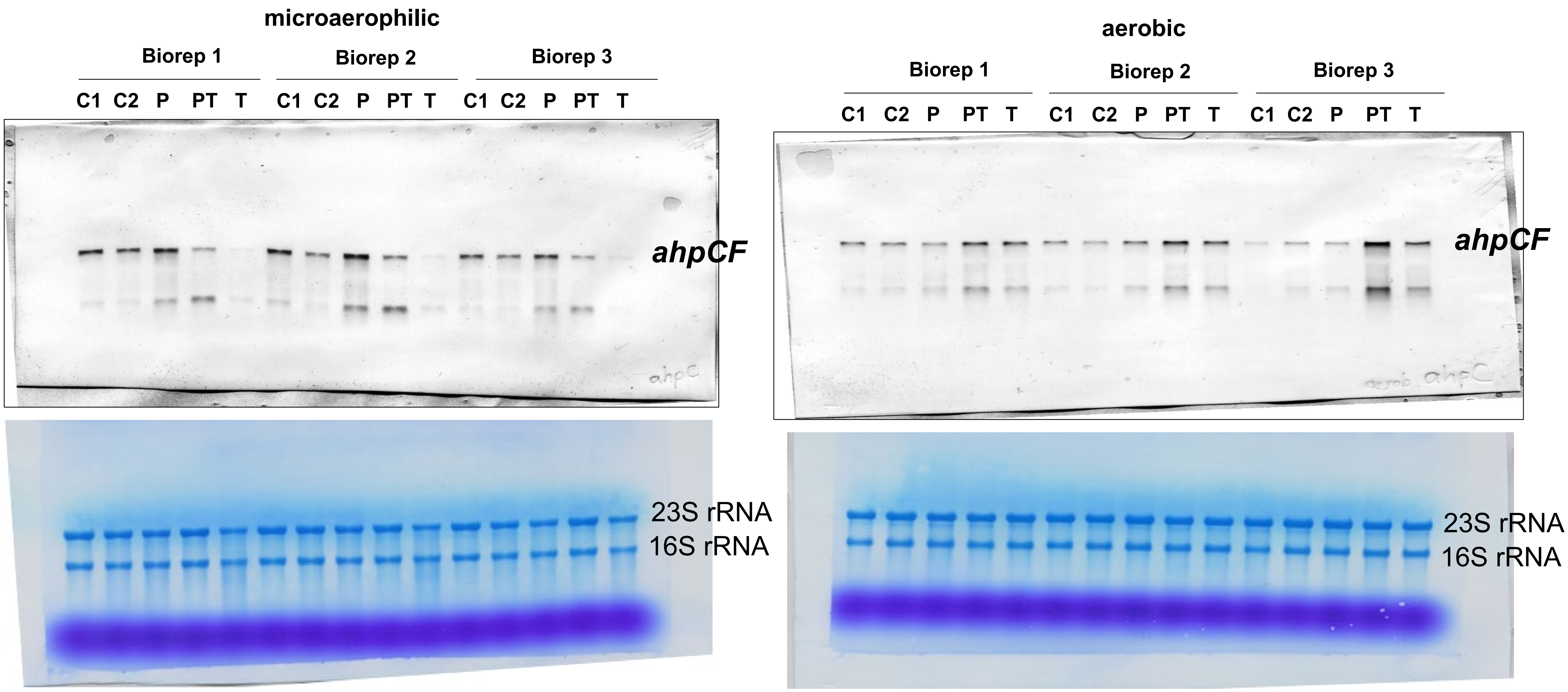

Fig. S10. A) The Northern blot images of priming experiments using the *ahpC* specific RNA probe show the *ahpCF* transcripts (images of Fig. 5B,C; 3 bioreplicates). **B)** The methylen blue stain of the Northern blots used as loading control for total RNA (images of Fig. 5B,C; 3 bioreplicates). Labelling of RNA samples is as above for the detection of the *ahpCF* transcript. The bands for the 16S and 23S rRNAs are indicated.

Fig. S11

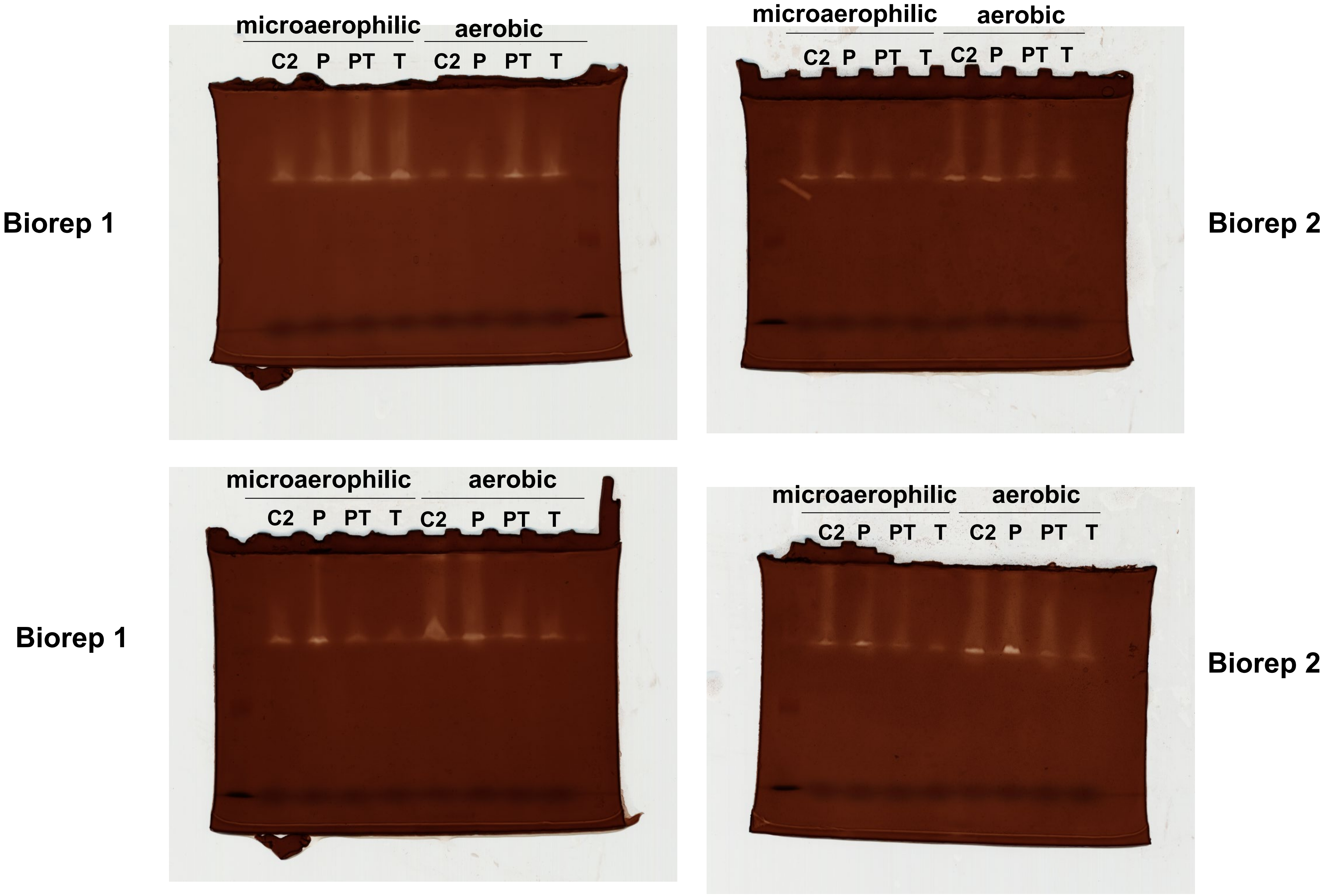

Fig. S11. Native gels of catalase stains used for KatA activity assays (images of Fig. 5F; 2 bioreplicates, 2 technical replicates)

Fig. S12

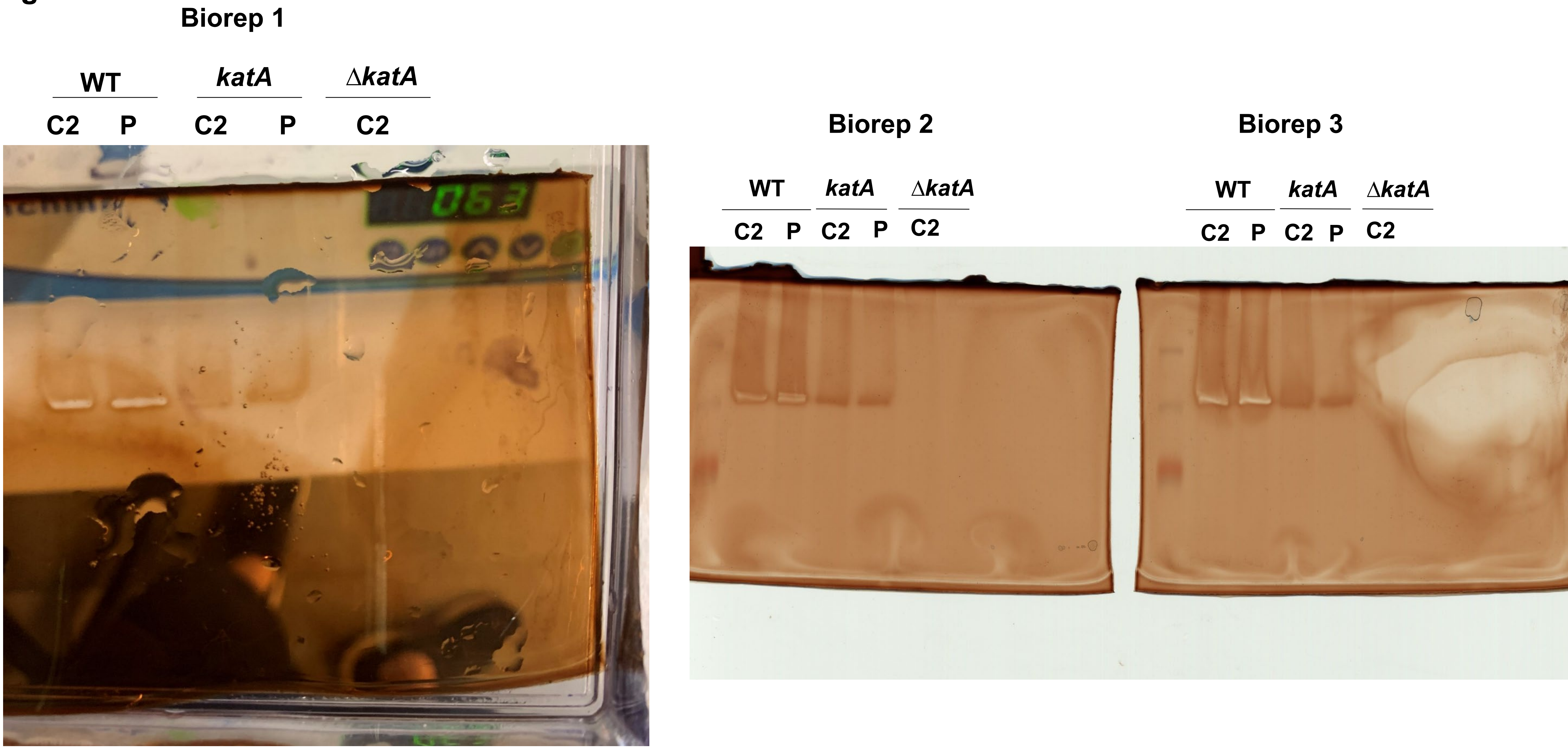

Fig. S12. Native gels of catalase stains used for KatA activity assays (images of Fig. S1D; 3 bioreplicates)

Fig. S13

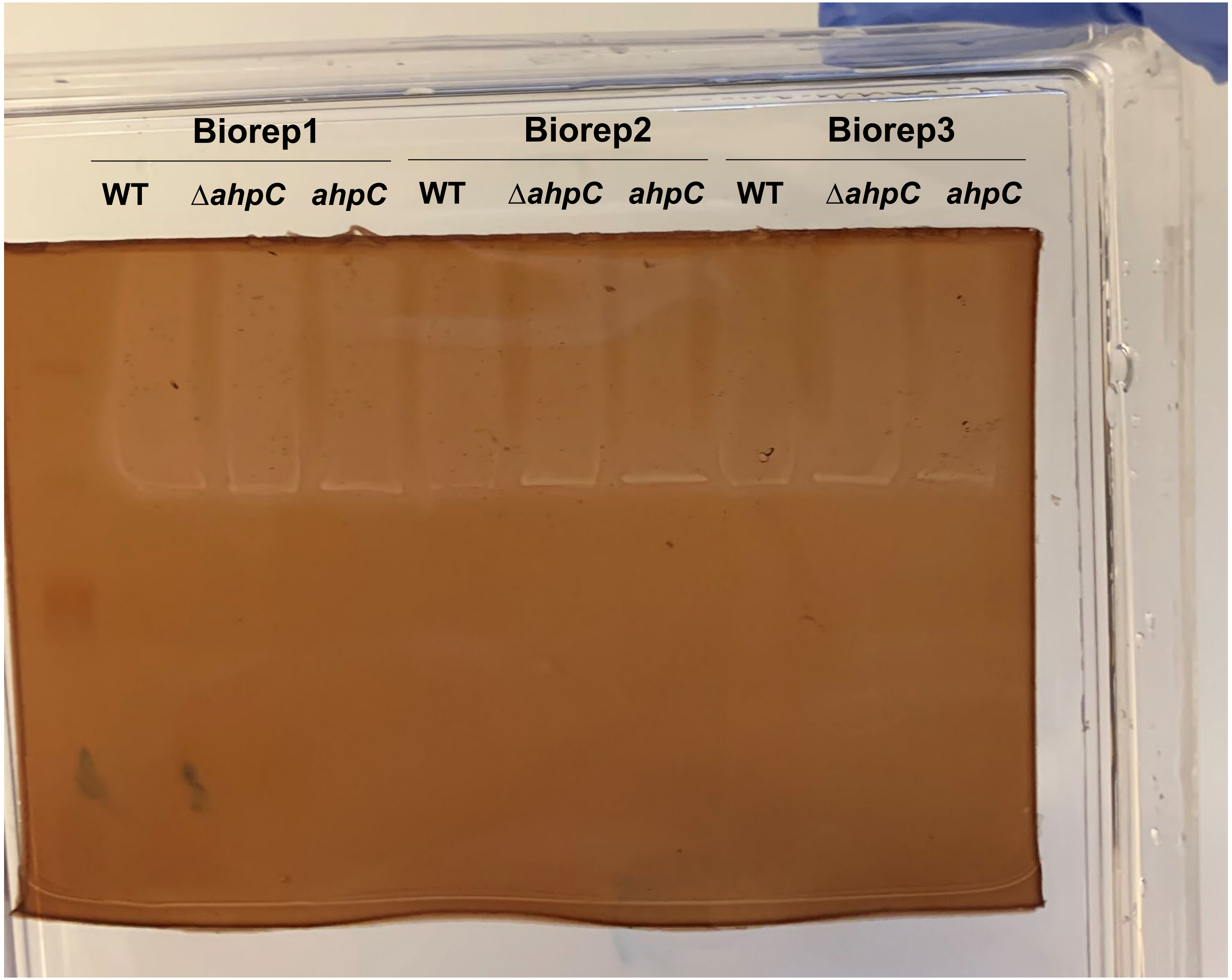

Fig. S13. Native gels of catalase stains used for KatA activity assays (images of Fig. S2B; 3 bioreplicates)
